# Facile access to deep red/near-infrared emissive AIEgens for efficient non-doped OLEDs†
†Electronic supplementary information (ESI) available. See DOI: 10.1039/c8sc01377b


**DOI:** 10.1039/c8sc01377b

**Published:** 2018-06-25

**Authors:** Will W. H. Lee, Zheng Zhao, Yuanjing Cai, Zeng Xu, Ying Yu, Yu Xiong, Ryan T. K. Kwok, Yue Chen, Nelson L. C. Leung, Dongge Ma, Jacky W. Y. Lam, Anjun Qin, Ben Zhong Tang

**Affiliations:** a Department of Chemistry , Hong Kong Branch of Chinese National Engineering Research Center for Tissue Restoration and Reconstruction , Institute of Molecular Functional Materials , Division of Life Science and Biomedical Engineering , State Key Laboratory of Nanoscience , The Hong Kong University of Science and Technology , Clear Water Bay , Kowloon , Hong Kong , China . Email: tangbenz@ust.hk; b HKUST-Shenzhen Research Institute , No. 9 Yuexing 1st RD, South Area, Hi-tech Park, Nanshan , Shenzhen 518057 , China; c NSFC Center for Luminescence from Molecular Aggregates , SCUT-HKUST Joint Research Laboratory , State Key Laboratory of Luminescent Materials and Devices , South China University of Technology , Guangzhou 510640 , China . Email: msdgma@scut.edu.cn

## Abstract

Notwithstanding the huge demand in bio-imaging and optoelectronics, the construction of highly emissive deep red/near infrared (DR/NIR) organic luminogens is still a big challenge because a narrow energy gap generally leads to low photoluminescence quantum yield. It is even more difficult to afford DR/NIR emitters in the solid state due to the aggregation caused quenching (ACQ) effect. In this work, we found that the direct attachment of a tetraphenylethylene substituted arylamine to the electron accepting 2,1,3-benzothiadiazole produces DR/NIR AIE luminogens with bright emission facilely and efficiently. And the emission wavelengths could be tuned from the red to the DR/NIR region by regulating the variety of the substituents. The long emission wavelength and high photoluminescence quantum yield of these AIEgens are ascribed to the effective intramolecular charge transfer and the suppressed intramolecular motion. Furthermore, non-doped OLEDs based on one of the AIEgens showed an EL emission at 684 nm with a large radiance of 5772 mW Sr^–1^ m^–2^ and an impressive external quantum efficiency (EQE) of 1.73%.

## Abstract 2

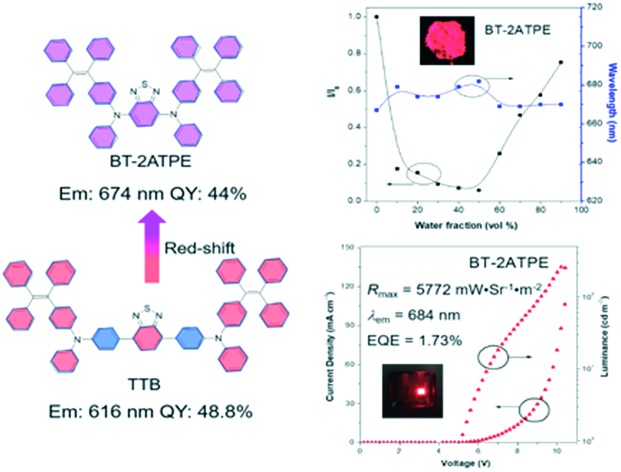
Design of an efficient DR/NIR solid emitter with a shortened conjugation length but long emission wavelength for non-doped NIR OLEDs.

## 1. Introduction

Advanced bio-imaging technology and optoelectronics have encouraged the rapid advancement of fluorescent materials with deep red/near-infrared (DR/NIR) emission (650–900 nm).^1^–^3^ Indeed, luminogens with DR/NIR emission could minimize the photo-damage to living cells, enhance the tissue penetration depth, and circumvent the spectral overlap with autofluorescence of bio-substrates.^1^ In terms of optoelectronic devices, red and DR/NIR emitters are essential components for constructing full color and NIR organic light-emitting diodes (OLEDs). Particularly, OLEDs with NIR emission are of crucial importance in optical communication, night-vision devices and information-secured displays.^2^,^3^ However, lower energy gaps usually lead to larger vibronic coupling between the ground and excited states, which detrimentally increases the radiationless decay rate of excitons and decreases the photoluminescence quantum yield (PLQY) of the luminogens, according to the energy gap law.^4^ In view of this, the design and synthesis of efficient DR/NIR emitters is still a huge challenge.

Much effort has been devoted to developing highly efficient emitters with DR/NIR emission, including transition-metal complexes (*e.g.*, Pt^2+^, Os^3+^ or Ir^3+^), lanthanide complexes, organic small molecules, and conjugated polymers.^5^–^7^ Amongst these materials, transition-metal complexes with phosphorescence achieve DR/NIR luminescence easily because the energy level of the triplet excited state is intrinsically lower than that of the singlet excited state. Several transition-metal complexes have exhibited emission peaks in the range of 700–900 nm and achieved high PLQYs of 15–82% and external quantum efficiencies (EQEs) of 3–24% in their OLED devices5a,b However, metal complexes are commonly associated with air- and moisture-instability, environmental toxicity and high cost. On the other hand, metal-free organic luminogens are arousing great research interest because they possess advantages of molecular diversity, low cost, and tunable energy gaps as well as the capability of mass production.^2^,^3^,^8^ Additionally, by taking advantage of the excited state with both local excitation and charge transfer (CT) features and regulating the orbital separation between the highest occupied molecular orbital (HOMO) and the lowest unoccupied molecular orbital (LUMO), new principles of hybridized local and charge transfer (HLCT) and thermally activated delayed fluorescence (TADF) have been proposed to break through the limit of 25% EQE in OLED devices.^2^,^3^ However, it is worth noting that for both HLCT and TADF characterized molecules, their design is still complex especially for the ones with DR/NIR emission as well as high PLQY. Furthermore, most of the organic luminogens with relatively planar structures suffer from aggregation-caused quenching (ACQ)^9^ due to their strong intermolecular π–π stacking resulting from the dipole–dipole/electrostatic interactions.^10^ This ACQ effect further intensifies the challenge of the molecular design of DR/NIR solid emitters.

Organic molecules aggregate naturally when they are fabricated as thin film devices. For conventional planar luminogens, aggregation is detrimental to light emission as a result of the ACQ effect. Because of this, organic emitters are typically used as dopants in OLED devices with precise control of the doping concentration to decrease the ACQ effect and suppress the serious efficiency roll-off caused by exciton annihilation at high current density.^11^,^12^ The doping strategy can partially address the problem of declined electroluminescence (EL) efficiency, but new problems emerge such as high cost of mass production, performance degradation by phase separation upon heating and complexity of technology due to addition of dopants. Thus, it is highly desirable to develop non-doped emitting systems.

In 2001, Tang and co-workers reported a phenomenon of aggregation-induced emission (AIE),^13^ which is exactly the opposite of the ACQ effect. AIE luminogens (AIEgens) generally exhibit weak/no luminescence in dilute solutions but emit intensely in the aggregated state. The efficient solid-state emission of AIEgens has been proved to be an effective approach to combat the ACQ effect and exciton annihilation of EL devices; thus, AIEgens are suitable for non-doped OLEDs.^14^ Thanks to the efforts of scientists, many AIEgens with high PLQY have been prepared and applied for OLED fabrication. So far, most of them have emitted efficiently in the short wavelength region (≤630 nm) while the ones with strong DR/NIR emission are still rare.^11^,^14^ Construction of donor (D)–acceptor (A) type π-conjugated structures is one of the most effective strategies to generate DR/NIR luminogens since the HOMO and LUMO energy levels and the energy gap can be finely tuned by selecting different D and A moieties.^1^–^3^,^9^ Nevertheless, the relatively twisted D–A structure of AIEgens makes it easier to access the non-radiative decay pathway by twisted intramolecular charge transfer (TICT) and molecular motion.9*a* Therefore, to achieve AIEgens with bright DR/NIR emission, it is favorable to rationally tune the intramolecular charge transfer (ICT) strength of D–A structures while suppressing the molecular motion as much as possible.

**Scheme 1:**
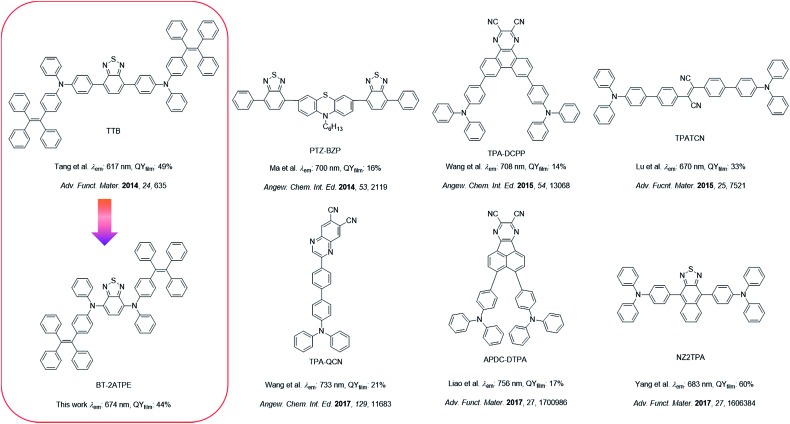
The design of BT-2ATPE and summary of the state-of-the-art NIR emitters.

2,1,3-Benzothiadiazole (BT) is a widely used building block to construct fluorescent compounds due to its π-extended structure and electron withdrawing properties.^15^ In our previous work, BT was used as a bridge to connect two tetraphenylethylene (TPE)-substituted diphenylamine moieties. The resulting AIEgen (TTB) exhibited an emission wavelength of 617 nm with a high PLQY of 49% (Scheme 1).9*c* Despite its high PLQY and wide applications in blood vascular imaging, long-term cell tracing and OLED devices, the synthesis and purification of TTB and its derivatives are tough and the corresponding emission wavelengths are rather short.9c–e,11a In this work, we observed that the direct attachment of TPE substituted aniline and naphthylamine (ATPE and NATPE) to BT by the Buchwald–Hartwig cross coupling reaction produced novel AIEgens of BT-2ATPE and BT-2NATPE with much longer absorption and emission wavelengths as well as comparable solid-state PLQYs (44% and 30%) to TTB, despite their shortened conjugation length. The underlying mechanism was investigated. These PLQY values are comparable with those of the latest developed high-performance DR/NIR emitters (Scheme 1).2a–c,3b–d,9c Furthermore, the Buchwald–Hartwig cross-coupling reaction can generate both mono- and di-substituted products with high yields by changing the ratio of reactants, enabling facile access to a series of symmetrical and asymmetrical AIEgens with tunable emissions in the red and NIR regions. Based on the bright and NIR emission of BT-2ATPE and BT-2NATPE, their non-doped NIR OLED devices were fabricated to evaluate their electroluminescence performance. Particularly, BT-2ATPE exhibited an EL wavelength of 684 nm with a high radiance of 5772 mW Sr^–1^ m^–2^ and an EQE of 1.73%, indicating the great potential of these AIEgens for non-doped NIR OLEDs.

## 2. Results and discussion

### 2.1. Synthesis and photophysical properties

The molecular design is shown in Scheme 1. As mentioned, our group previously reported on TTB, which showed a high PLQY of 49% but a rather short emission wavelength of 617 nm as well as tedious synthesis and purification. On the other hand, Yang *et al.* reported that the di-substitution of the BT moiety with diphenylamine can generate a longer emission wavelength of 648 nm than that of TTB despite its ACQ characteristic.9*f* Mataka and coworkers also demonstrated that the π-spacer length in between the donor and the acceptor is inversely proportional to their emission wavelength.9*g* We thus wondered whether we could simplify the molecular structure of TTB by direct attachment of ATPE or NATPE to afford molecules with a small size but longer emission wavelength and high PLQY.

**Scheme 2:**
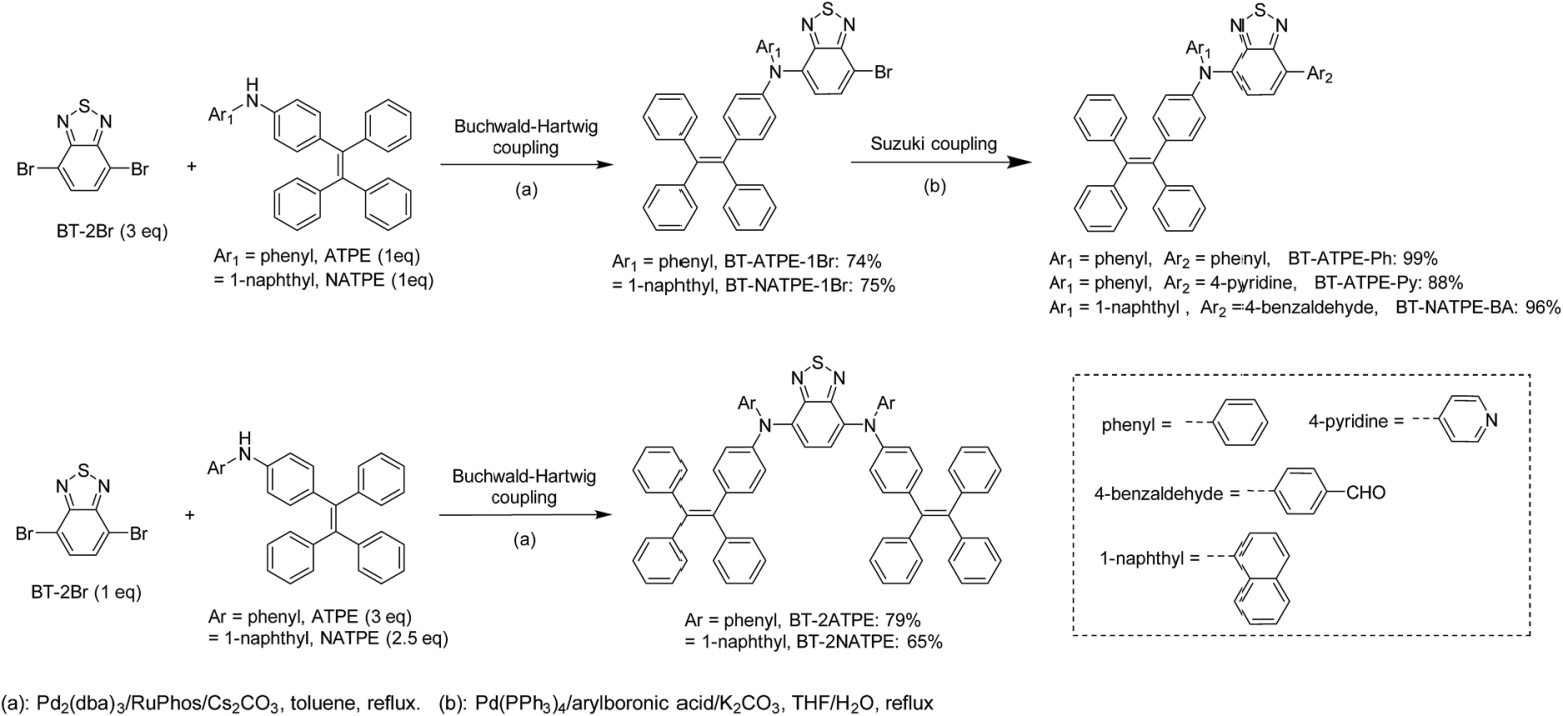
The synthetic route for BT-2ATPE, BT-2NATPE and other derivatives.

The synthetic routes for the target compounds are presented in Scheme 2. The synthesis started from the coupling of 4,7-dibromobenzo[*c*][1,2,5]thiadiazole (BT-2Br) with *N*-phenyl-4-(1,2,2-triphenylvinyl)aniline (ATPE) or *N*-(4-(1,2,2-triphenylvinyl)phenyl)naphthalen-1-amine (NATPE).9*c* At first, the commonly used Buchwald–Hartwig conditions employing Pd(OAc)_2_ and P(*t*-Bu)_3_ as the catalyst and ligand, respectively, were implemented, which unfortunately resulted in low yield. This could possibly be due to the steric hindrance of the bulky ATPE or NATPE hampering the reactivity. After preliminary screening, the coupling was implemented by employing Pd_2_(dba)_3_ and RuPhos as the catalyst and ligand, respectively, since this combination was demonstrated to be more suitable for both aromatic and aliphatic secondary amine substrates.^16^ The reaction proceeded smoothly, affording BT-2ATPE and BT-2NATPE as purple-red solids in high and moderate yields of 79% and 65%, respectively. The side products of the reactions were the mono-substituted products BT-ATPE-1Br/BT-NATPE-1Br, which could be utilized to give access to asymmetrical AIEgens. The mono-substituted products BT-ATPE-1Br/BT-NATPE-1Br could also be efficiently prepared by changing the ratio of BT-2Br and ATPE/NATPE to 3 : 1. Suzuki couplings of BT-ATPE-1Br/BT-NATPE-1Br with different boronic acids were very efficient, affording asymmetrical products BT-ATPE-Ph, BT-ATPE-Py, and BT-NATPE-BA in high yields (99%, 88% and 96%, respectively). These asymmetrical products can possibly act as intermediates to construct bio-imaging agents or other new π-functional materials. All the compounds and intermediates were fully characterized by ^1^H NMR, ^13^C NMR and high-resolution mass spectrometry with satisfactory analysis results (Fig. S1–S21†).

**Fig. 1:**
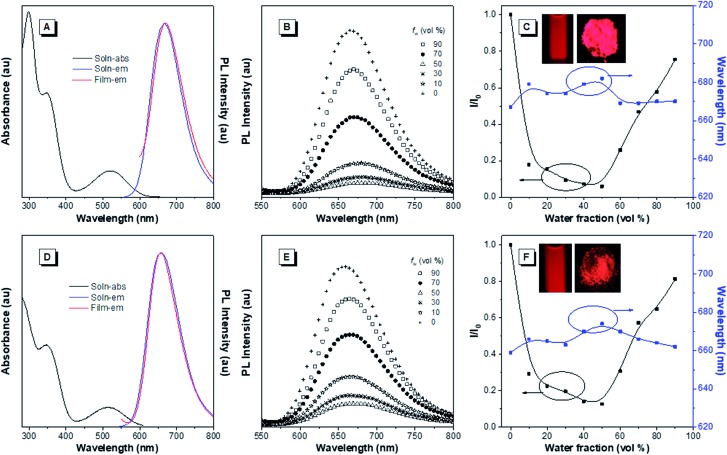
(A and D) Absorption and PL spectra of (A) BT-2ATPE and (D) BT-2NATPE in dilute THF solutions (10 μM) and thin films. (B and E) PL spectra of (B) BT-2ATPE and (E) BT-2NATPE in THF/H_2_O mixtures with different water fractions (*f*_w_). Concentration: 10 μM; *λ*_ex_: 520 nm. (C and F) The plots of the emission maximum and the relative emission intensity (*I*/*I*_0_) *versus* the composition of the aqueous mixture of (C) BT-2ATPE and (F) BT-2NATPE. *I*_0_ = PL intensity in pure THF. Inset: fluorescence photographs of BT-2ATPE and BT-2NATPE in dilute THF solutions and as powder taken under 365 nm UV irradiation.

**Fig. 2:**
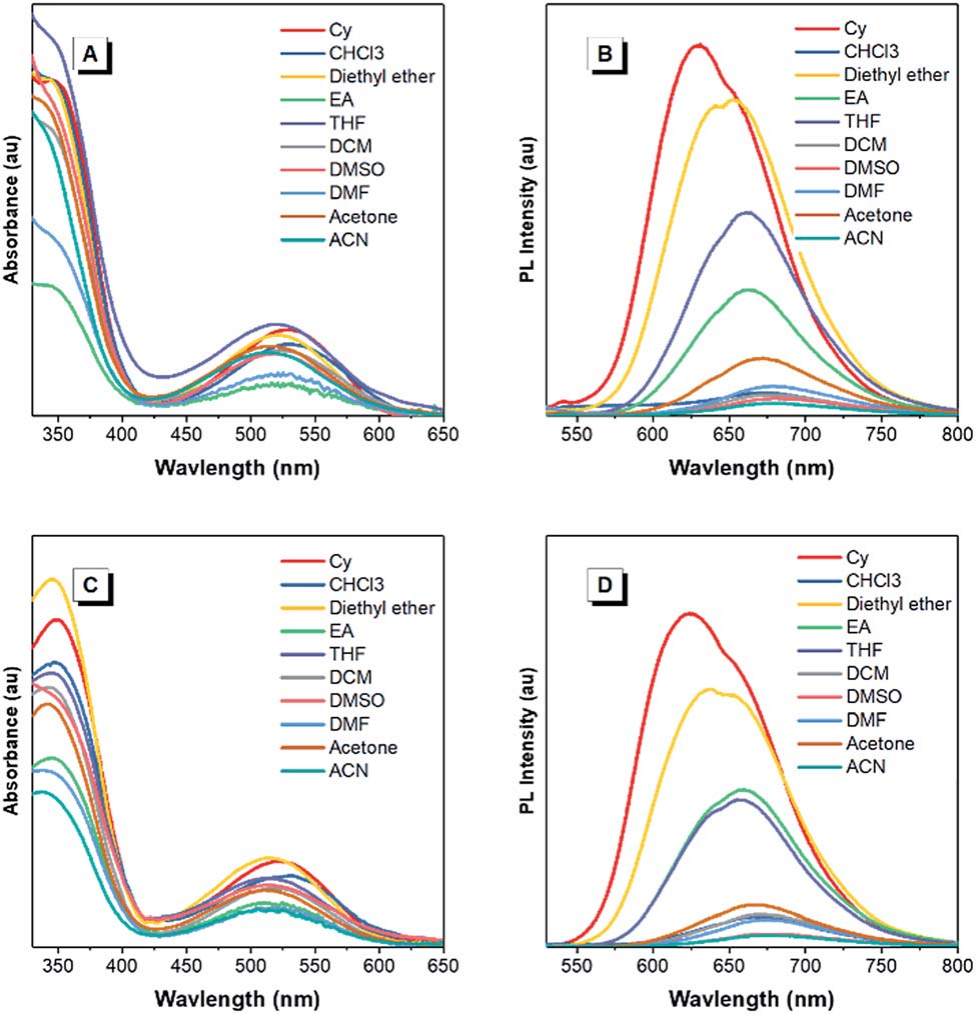
(A and C) Absorption and (B and D) PL spectra of (A and B) BT-2ATPE and (C and D) BT-2NATPE in solvents with different polarities. Concentration: 10 μM. The absorption maximum of each solution was chosen as its excitation wavelength.

The optical properties of the asymmetrical and symmetrical products were systematically studied by UV-vis absorption and photoluminescence (PL) spectroscopies (Fig. 1 and S22†). As shown in Fig. S22 and Table S1,† BT-ATPE-Ph, BT-ATPE-Py and BT-NATPE-BA exhibited similar absorption spectra with two absorption bands at around 350 nm and 485 nm, respectively. In dilute THF solution, BT-ATPE-Ph, BT-ATPE-Py and BT-NATPE-BA showed dim emission with emission maxima at 620 nm, 615 nm and 625 nm, respectively, while in the aggregated or solid state, all three compounds showed enhanced emission (*Φ*_F,solid,BT-ATPE-Ph_ = 19%, *Φ*_F,solid,BT-ATPE-Py_ = 8%, *Φ*_F,solid,BT-NATPE-BA_ = 8%) with the emission of BT-ATPE-Ph exhibiting a slight red-shift (*λ*_em,max_ = 635 nm) but those of BT-ATPE-Py (*λ*_em,max_ = 660 nm) and BT-NATPE-BA (*λ*_em,max_ = 650 nm) red-shifting a lot relative to their solutions (Fig. S22†). The larger PL red-shift of BATPE-Py and BT-NATPE-BA than BT-ATPE-Ph was possibly due to their improved planarity and the stronger intermolecular interaction in the solid state.^17^ The symmetrical products (BT-2ATPE and BT-2NATPE) generally exhibited longer absorption and emission wavelengths than the asymmetrical products (BT-ATPE-Ph, BT-ATPE-Py and BT-NATPE-BA) due to their larger π-conjugation. As shown in Fig. 1 and Table 1, BT-2ATPE in dilute THF solution exhibited two absorption peaks at 346/522 nm. The absorption profile of BT-2NATPE was almost identical to that of BT-2ATPE. The peak at the shorter wavelength should be ascribed to a π–π* transition, and the longer one was associated with the ICT from the arylamine segment to the BT moiety. Compared to previously reported AIEgens TTB and TNB (Fig. S23†), which possess similar chemical structures to BT-2ATPE and BT-2NATPE but with two additional phenyl rings in between the BT and amine units,9*c* BT-2ATPE and BT-2NATPE showed much redder absorptions (522 nm and 520 nm) than TTB and TNB (471 nm and 470 nm). The red-shift of the absorptions of BT-2ATPE and BT-2NATPE should be ascribed to their stronger ICT effect as compared to TTB and TNB because the electron donating nitrogens in TTB and TNB had been spatially separated from the electron accepting BT moiety by the additional phenyl rings; more phenyl ring substitution would lower the electron density of the nitrogen, thus weakening the ICT. In dilute THF solution, both BT-2ATPE and BT-2NATPE showed bright emissions with emission peaks at 666 nm and 658 nm, respectively, which are much redder than the emissions of TTB (616 nm) and TNB (613 nm). This indicates that direct attachment of the electron donor to the electron acceptor is a more efficient approach to extend both absorption and emission, due to the more effective ICT effect. From solution to thin film, strong PL signals of BT-2ATPE and BT-2NATPE were maintained with the emission maximum of BT-2ATPE slightly shifted to 674 nm while that of BT-2NATPE having no shift (658 nm). The slight changes in the emission properties of BT-2ATPE and BT-2NATPE from solution to thin film imply that the molecular deformation of BT-2ATPE and BT-2NATPE in the excited state is inactive, possibly due to the good electron conjugation and the large steric hindrance between the arylamine and the BT unit, which limit the rotors from free rotation or vibration.^18^ Consequently, the non-radiative decay pathway of the exciton deactivation was greatly suppressed and thus BT-2ATPE and BT-2NATPE fluoresced brightly. The AIE properties of BT-2ATPE and BT-2NATPE were investigated by studying their light emitting behaviors in THF/water mixtures with different water fractions (*f*_w_). Upon adding water into the THF solutions of BT-2ATPE and BT-2NATPE, their PL intensities gradually decreased at first (*f*_w_: 0–40%) but started to increase when the *f*_w_ exceeded 50% (Fig. 1). Meanwhile, the emission maxima changed within a magnitude of 10 nm. This PL profile of BT-2ATPE and BT-2NATPE indicates that they exhibit both AIE and TICT features.^19^ To further evaluate their TICT effect, we recorded their absorption and emission spectra in different solvents. As shown in Fig. 2, BT-2ATPE and BT-2NATPE shared similar absorption profiles and their absorption maxima changed only a little with the variation of solvent polarity. In sharp contrast, their luminescence behaviors changed more obviously. In nonpolar cyclohexane (Cy), BT-2ATPE and BT-2NATPE showed strong red emissions with emission wavelengths of 631 nm and 624 nm, respectively. Their emission colors shifted to deep red in polar solvent (THF), showing emission maxima of 666 nm and 658 nm. In solvents with strong polarity such as DMSO, their emission maxima changed slightly (680 nm and 658 nm) but their luminescence became very dim. Their photophysical responses to solvent polarity unambiguously demonstrated the TICT characteristics of BT-2ATPE and BT-2NATPE. The Lippert–Mataga equation has been commonly used to study the relationship between the Stokes shift and the solvent polarity parameter (Δ*f*) to evaluate the strength of the TICT effect.9a,^19^ From the plot of the Lippert–Mataga equation (Fig. S24†), BT-2ATPE and BT-2NATPE exhibited relatively small slopes of 5510 cm^–1^ and 5854 cm^–1^, respectively, in comparison with their analogue TTB (9807 cm^–1^) and other typical compounds with strong TICT properties.^2^,9a,^20^ Plots of the Stokes shift against the solvent polarity scale *E*_T_(30), another method that correlates the Stokes shift and solvent polarity, also showed the same correlation (Fig. S24†).^21^ The change of dipole moments from the ground state to the excited state can also characterize the TICT effect; we thus took TNB and BT-2NATPE as an example and calculated and compared the change of their dipole moments in the ground state and excited state. TNB showed dipole moments of 1.51 D and 1.05 D in the ground state and excited state, respectively, while BT-2NATPE exhibited dipole moments of 3.22 D and 2.69 D in the ground state and excited state, respectively. The dipole moment change was calculated using the formula of Δ*μ* = (|μ* – *μ*|)/*μ*. The results indicate that TNB exhibits a dipole moment change of 0.30 D, which is larger than that of BT-2NATPE (0.16 D); therefore, BT-2NATPE shows a relatively weaker TICT effect. The phenomenon of BT-2ATPE and BT-2NATPE possessing a weaker TICT effect in spite of the more efficient ICT possibly originated from their good intramolecular D–A conjugation and the inactive intramolecular deformation which rigidify the molecular conformation. In the absence of the phenyl ring spacers, the TPE moieties of BT-2ATPE and BT-2NATPE are situated closer to the BT core, which should impose stronger steric hindrance, thus restricting the free rotation of the C–N bond between the D and A units, resulting in a weaker TICT effect. Furthermore, the PLQYs of BT-2ATPE and BT-2NATPE in solution and thin film were determined using an integrating sphere. In dilute THF solutions, both BT-2ATPE and BT-2NATPE exhibited a PLQY of 32%. More importantly, the PLQYs of BT-2ATPE and BT-2NATPE in thin films were as high as 44% and 30%, respectively, inspiring us to explore their application in OLED devices.

**Table 1:** Optical and thermal properties of BT-2ATPE and BT-2NATPE^*a*^

	*λ*_abs_ (nm)	*λ*_em_ (nm)		*Φ* (%)		HOMO (eV)	LUMO (eV)	*T*_d_/*T*_g_ (°C)
		Soln	Film	Soln	Film			
BT-2ATPE	364, 522	666	674	32	44	–5.0	–2.88	480/135, 234
BT-2NATPE	346, 520	658	658	32	30	–5.1	–2.81	488/163

^*a*^Abbreviations: *λ*_abs_ = absorption maximum; *λ*_em_ = emission maximum; HOMO = highest occupied molecular; LUMO = lowest unoccupied molecular orbital; *T*_d_ = temperature for 5% weight loss measured by TGA; *T*_g_ = glass transition temperature determined by DSC; soln = chloroform.

### 2.2. Theoretical calculation, and electrochemical and thermal properties

**Fig. 3:**
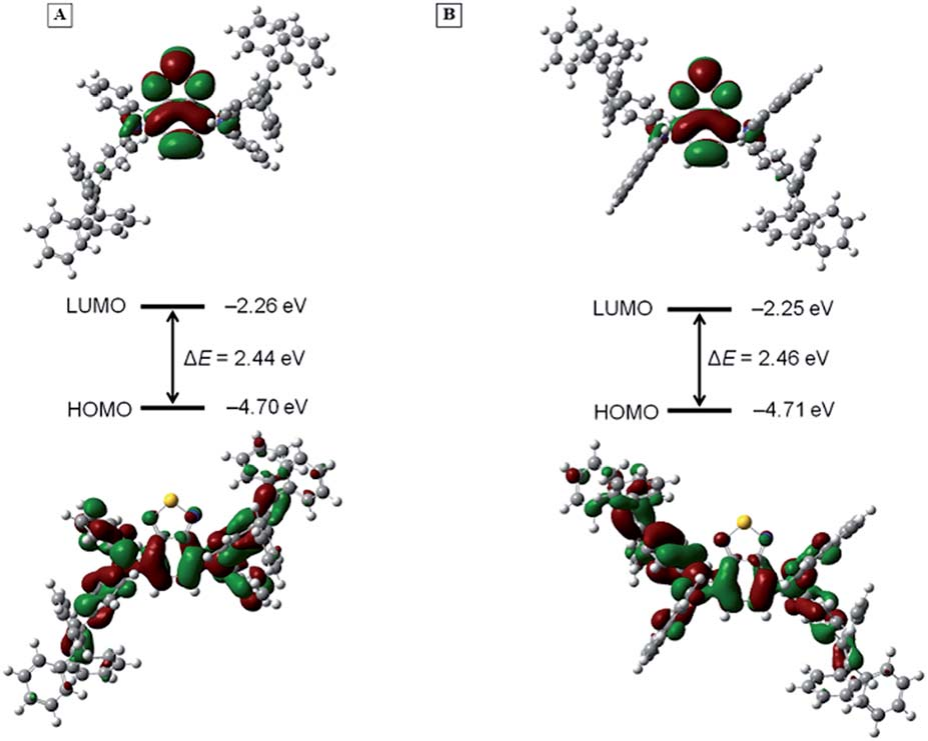
The electron density distribution of frontier orbitals and energy levels of (A) BT-2ATPE and (B) BT-2NATPE calculated using DFT at the B3LYP/6-31G(d,p) level. Abbreviations: LUMO = lowest unoccupied molecular orbital; HOMO = highest occupied molecular orbital; Δ*E* = energy gap.

To further interpret the photophysical properties of BT-2ATPE and BT-2NATPE at the molecular level, density functional theory (DFT) calculation was carried out using the Gaussian 09 suite of programs. The nonlocal density functional of B3LYP with 6-31G(d,p) basis sets was used for the calculation. The optimized molecular geometries shown in Fig. S25† demonstrated that both BT-2ATPE and BT-2NATPE possess twisted configurations with similar torsion angles of 33.44–54.62° between the BT unit and the phenyl rings of the adjacent arylamines. These twisted conformations of the two compounds were derived from the intrinsic twisting structure of the TPE segments and the large steric hindrance between the BT unit and the phenyl rings of the adjacent arylamine. Such bulky conformations and crowded structures of the two molecules not only prohibit close intermolecular packing but also forbid intramolecular motion, both of which contribute to the bright solid emission.^18^Fig. 3 shows the frontier orbital distribution of the two molecules. For both molecules, the electron cloud of the HOMOs is distributed along the central BT moiety and the arylamine segments, indicating good intramolecular conjugation. Meanwhile, the LUMOs were mainly dominated by the orbitals from the BT core and the nitrogen atom, suggesting an intramolecular charge transfer (ICT) from the arylamine segments to the BT unit. The twisted configurations and the electron density difference are in well accordance with their TICT characteristic. The HOMO and LUMO energy levels of BT-2ATPE estimated using DFT were –4.70 eV and –2.26 eV, respectively, which were comparable with that of BT-2NATPE (HOMO = –4.71 eV and LUMO = –2.25 eV). The smaller band gap of BT-2ATPE (2.44 eV) than BT-2NATPE (2.46 eV) was consistent with the experimental data.

Besides DFT calculation, the HOMO and LUMO energy levels of the two molecules were also determined by cyclic voltammetry (CV) measurements according to the equations HOMO = –4.4 – *E*1/2ox/*E*onsetox and LUMO = –4.4 – *E*1/2red/*E*onsetred.^22^ As shown in Fig. S26,† BT-2ATPE exhibited two irreversible oxidative waves with an onset voltage of *E*onsetox = 0.60 V and reversible reductive waves with a half-wave potential of *E*1/2red = –1.52 V, while BT-2NATPE exhibited an irreversible reductive wave (*E*onsetred = –1.59 V) and two reversible oxidation waves with a half-wave potential of *E*1/2ox1 = –0.67 V and *E*1/2ox2 = –0.99 V, respectively. The CV results suggest that the HOMO and LUMO energy levels of BT-2ATPE are –5.0 eV and –2.88 eV, while those of BT-2NATPE are –5.1 eV and –2.81 eV. Apparently, both BT-2ATPE and BT-2NATPE are suitable for hole transporting.

Since the thermal properties of the emitting materials are important parameters for device fabrication, we investigated their thermal properties by thermogravimetric analysis (TGA) and differential scanning calorimetry (DSC). The TGA results showed that both BT-2ATPE and BT-2NATPE were thermally stable, losing merely 5% of their weight at high temperatures of 340 °C and 369 °C, respectively (Fig. S27†). The DSC thermogram of BT-2ATPE showed a strong and sharp endothermic peak at 279 °C in the first heating cycle, corresponding to its melting point. Because the first heating process can destroy the orderly alignment of the molecules, two additional phase transition peaks at 135 °C and 234 °C were observed besides the melting point in the second heating cycle (Fig. S28†). It is noteworthy that AIEgens generally possess a twisted and flexible molecular conformation, which endows them with polymorphism characteristics.8*b* Therefore, in most cases, only broad and weak phase transition temperatures could be observed during the heating cycles.8b,11c In this case, the strong and sharp phase transition peak of BT-2ATPE implies its particularly rigid structure. For BT-2NATPE, only one glass transition temperature at about 163 °C was observed. The high phase transition temperatures of BT-2ATPE and BT-2NATPE are suggestive of their high morphological stability.

### 2.3. Device characterization

**Fig. 4:**
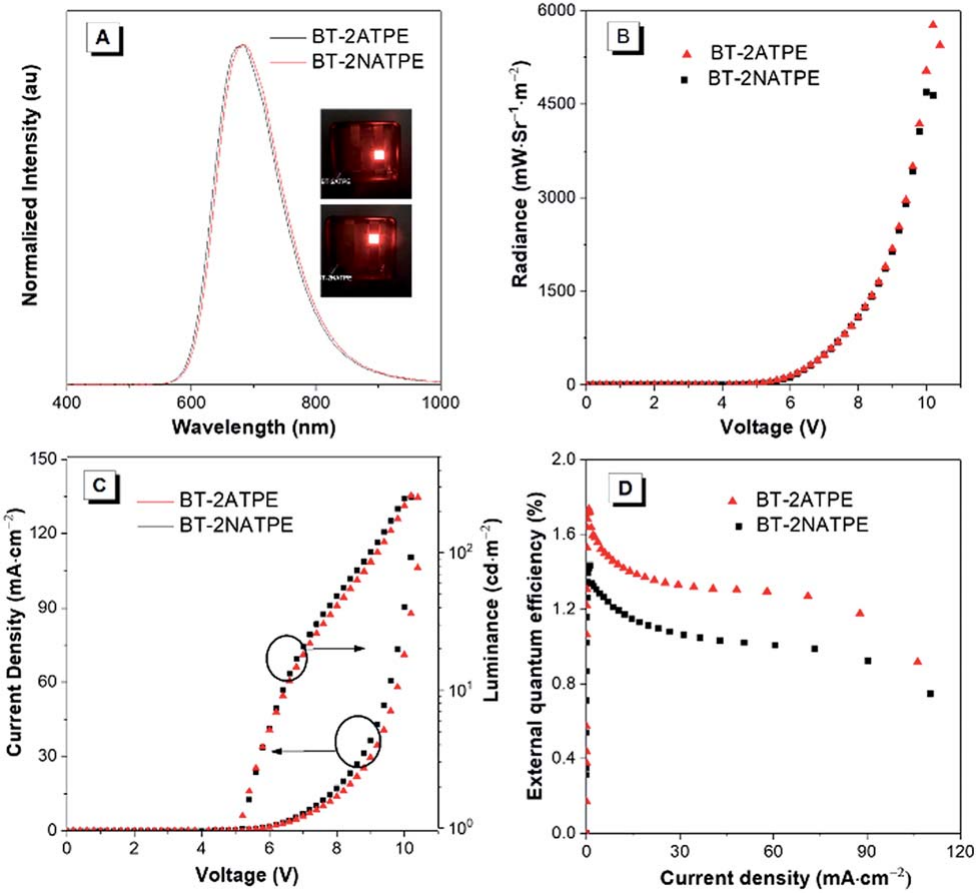
(A) EL spectrum of BT-2ATPE and BT-2NATPE. Inset: photos of the devices. (B) Maximum radiance of BT-2ATPE and BT-2NATPE. (C) Current density–voltage–luminance characteristics. (D) The plot of external quantum efficiency against the applied current.

The high solid state PLQYs and good thermal and morphological stabilities of BT-2ATPE and BT-2NATPE encouraged us to investigate their EL properties. The non-doped EL devices with the configuration of indium tin oxide (ITO)/1,4,5,8,9,11-hexaazatriphenylenehexacarbonitrile (HATCN) (5 nm)/4,4′-cyclohexylidenebis[*N*,*N*-bis(4-methylphenyl)benzenamine] (TAPC) (60 nm)/tris(4-carbazoyl-9-ylphenyl)amine (TCTA) (5 nm)/BT-2ATPE or BT-2NATPE (20 nm)/Bphen (55 nm)/Liq (2 nm)/Al were fabricated by a vapor deposition process, in which BT-2ATPE or BT-2NATPE served as the emitter layer, HATCN worked as the hole injection layer, TCTA was the hole transporting layer, and Bphen functioned as the electron-transporting material. The EL spectra are shown in Fig. 4 and the device performances are summarized in Table S2.† The EL maxima of the non-doped devices based on BT-2ATPE and BT-2NATPE were located at 684 nm and 682 nm, respectively, which were slightly red-shifted from the PL maxima of their solid films (674 nm and 658 nm). The Commission Internationale de L'Eclairage (CIE) coordinates of BT-2ATPE and BT-2NATPE were (0.692, 0.305) and (0.688, 0.308), respectively (Fig. S29†). Both devices turned on at a voltage of 4.2 V. Additionally, the EL spectra did not show any changes upon increasing the driving bias voltages, demonstrating their stability (Fig. S30†). The devices of BT-2ATPE and BT-2NATPE exhibited large radiances of 5772 mW Sr^–1^ m^–2^ and 4692 mW Sr^–1^ m^–2^, respectively. In addition, the device of BT-2ATPE possessed a relatively higher EQE of 1.73% than BT-2NATPE (1.43%). Upon increasing the current density to 10 mA cm^–2^, the EQE of the BT-2ATPE device decreased to 1.44%, losing 16.7% of its maximum value, indicating a relatively low EQE roll-off. In comparison with the several reported NIR OLEDs which generally exhibited EQEs over 2% for their non-doped devices,^2^,3b,c the EQE of BT-2ATPE and BT-2NATPE may not be so impressive since they can only utilize the singlet excitons for emission. However, the design of BT-2ATPE and BT-2NATPE provides the advantages of simplicity and compatibility. We believe that with the continuous exploration of this molecular design strategy and by integrating it with other design strategies such as HLCT and TADF, better DR/NIR emitting fluorophores can be facilely afforded.

## 3. Conclusion

In summary, a series of AIEgens with tunable DR/NIR emissions and varied functional groups have been facilely synthesized by the direct coupling of a secondary arylamine donor with a commercially available BT acceptor. In particular, BT-2ATPE and BT-2NATPE exhibited emission maxima of 674 nm and 658 nm and achieved solid state PLQYs of 44% and 30%, respectively. In comparison with their structural analogues TTB and TNB, BT-2ATPE and BT-2NATPE showed much redder absorption and emission despite their shortened π-conjugation lengths. The longer absorptions, DR/NIR emissions and high PLQYs of BT-2ATPE and BT-2NATPE are ascribed to their strong ICT and rigid molecular structures. Therefore, this work demonstrates a facile and effective strategy to afford highly emissive DR/NIR AIEgens. BT-2ATPE and BT-2NATPE were applied to fabricate non-doped OLED devices and their device performances were evaluated. The BT-2ATPE device exhibited a large radiance of 5772 mW Sr^–1^ m^–2^ with a high EQE of 1.73%. This study not only presented an effective way to access DR/NIR AIEgens but also demonstrated their potential in non-doped NIR OLED devices.

## Conflicts of interest

The authors declare no conflicts of interest.

## Supplementary Material

Supplementary information
