# Supplementary material for: Facile access to deep red/near-infrared emissive AIEgens for efficient non-doped OLEDs
Source: Chem Sci. 2018 Jun 25;9(28):6118–25. doi: 10.1039/c8sc01377b (PMC6118221; doi:10.1039/c8sc01377b)
Supplement: Supplementary file 1 [file SC-009-C8SC01377B-s001.pdf]

Electronic Supplementary Material (ESI) for Chemical Science.
This journal is © The Royal Society of Chemistry 2018

Supporting Information

Facile Access to Deep red/Near-infrared Emissive AIEgens for Efficient Non-doped OLEDs

Will W. H. Lee, Zheng Zhao, Yuanjing Cai, Zeng Xu, Ying Yu, Yu Xiong, Ryan T. K. Kwok, Yue Chen, Nelson L. C. Leung, Dongge Ma Jacky W. Y. Lam, Anjun Qin, and Ben Zhong Tang**

Dr. Z. Zhao, Dr. Y. Xiong, Prof. B. Z. Tang
HKUST-Shenzhen Research Institute, No. 9 Yuexing 1st RD, South Area, Hi-tech Park, Nanshan, Shenzhen 518057, China
E-mail: tangbenz@ust.hk
W. W. H. Lee, Dr. Z. Zhao, Y. Yu, Dr. R. T. K. Kwok, N. L. C. Leung, Dr. J. W. Y. Lam, Prof. B. Z. Tang
Department of Chemistry, Hong Kong Branch of Chinese National Engineering Research Center for Tissue Restoration and Reconstruction, Institute of Molecular Functional Materials, Division of Life Science and Biomedical Engineering, State Key Laboratory of Nanoscience, The Hong Kong University of Science and Technology, Clear Water Bay, Kowloon, Hong Kong, China.
Dr. Y. Cai, Z. Xu, Prof. D. Ma, Prof. A. Qin, Prof. B. Z. Tang
NSFC Center for Luminescence from Molecular Aggregates, SCUT-HKUST Joint Research Laboratory, State Key Laboratory of Luminescent Materials and Devices, South China University of Technology, Guangzhou 510640, China.
E-mail: msdgma@scut.edu.cn

Experimental Section

Materials and Characterization

N-phenyl-4-(1,2,2-triphenylvinyl)aniline (ATPE) and N-(4-(1,2,2-triphenylvinyl)phenyl)naphthalen-1-amine (NATPE) were synthesized according to literature method, with TPE-Br purchased from AIEgen Biotech Co., Limited. All the other chemicals were purchased from Sigma-Aldrich or J&K and used as received without further purification. Toluene and THF were distilled from sodium-benzophenone under dry nitrogen immediately prior to use. ^{1}H and ^{13}C NMR spectra were measured on a Bruker AVII 400 NMR spectrometer using tetramethylsilane (TMS) as internal reference. High-resolution mass spectra (HRMS) were recorded using a Finnigan MAT TSQ 7000 Mass Spectrometer System operated in a MALDI-TOF mode. Absorption spectra were measured on a Milton Roy Spectronic 3000 Array spectrophotometer. Steady-state photoluminescence (PL) spectra were measured on a Perkin-Elmer spectrofluorometer LS 55. And PLQY was determined by an Quanta-φ integrating sphere. TGA measurements were carried out on a TA Q5000 instruments under a dry nitrogen flow at a heating rate of 10 °C/min, heating from room temperature to 800 °C. DSC analyses were performed on a TA Q1000 instrument under a nitrogen atmosphere at a heating (cooling) scan rate of 10 °C/min (rt−400 °C). Electrochemical measurements were performed on a CHI610D electrochemical workstation in a three-electrode cell using a platinum button as working electrode, a platinum wire as counter electrode and a saturated calomel electrode as reference electrode in CH_2Cl_2 solution with 0.1 M $Bu_4N^+ PF_6^-$ at a scan rate of 100 mV/s and ferrocene as internal standard.

Synthesis of BT-2ATPE

To a mixture of ATPE (508 mg, 1.2 mmol), 4,7-dibromobenzo[c]-1,2,5-thiadiazole (118 mg, 0.4 mmol), Cs_2CO_3 (430 mg, 1.2 mmol), $Pd_2(dba)_3$ (37 mg, 0.04 mmol) and RuPhos (37mg, 0.08mmol) under N_2, distilled toluene (8 mL) was added. The mixture was stirred in room temperature for 30 min before brought to reflux for 17 h. The reaction mixture was cooled to room temperature and extracted using water and DCM. The organic layer was collected and after solvent evaporation, the crude product was purified by silica-gel column chromatography using DCM/hexane (v/v =1:4 to 1:1) as eluent to afford BT-2ATPE as purple-red solid (310 mg, 79.1%). Further purification was done by recrystallization using DCM and MeOH.

^{1}H NMR (400 MHz, CD_2Cl_2), δ (ppm): 7.23 (t, 4H), 7.18-7.00 (m, 38H), 6.876 (dt, 4H), 6.753 (dt, 4H). ^{13}C NMR (100 MHz, CD_2Cl_2), δ (ppm): 152.6, 147.6, 146.1, 144.2, 144.0, 143.8, 140.9, 140.8, 138.5, 135.8, 132.3, 131.6, 131.6, 131.5, 129.3, 127.9, 127.8, 126.6,

126.6, 126.5, 125.0, 123.9, 123.2, 122.6. HRMS (MALDI-TOF): *m/z*: $[M]^+$ calcd for $C_{70}H_{50}N_4S$, 978.3756, found 978.3795.

Synthesis of BT-2NATPE

To a mixture of NATPE (712 mg, 1.5 mmol), 4,7-dibromobenzo[c]-1,2,5-thiadiazole (174 mg, 0.6 mmol), Cs_2CO_3 (600 mg, 1.8 mmol), $Pd_2(dba)_3$ (30 mg, 0.03 mmol) and RuPhos (30mg, 0.06mmol) under N_2, distilled toluene (30 mL) was added. The mixture was stirred in room temperature for 30 min and then at 110 °C overnight. After cooling to room temperature, the mixture was washed with water twice and extracted with dichloromethane and then purified by silica-gel column chromatography using DCM/hexane (v/v =1:4 to 2:1) as eluent to afford compound BT-2NATPE as purple solid (412 mg, 64.6%). Further purification was done by recrystallization using DCM and MeOH.

^1H NMR (400 MHz, $CDCl_3$), δ (ppm):7.908 (d, 2H), 7.846 (d, 2H), 7.711 (d, 2H), 7.466-7.426 (m, 2H), 7.381 (t, 2H), 7.349-7.308 (m, 2H), 7.240 (dd, 2H), 7.142-6.979 (m, 30H), 6.825 (s, 2H), 6.789 (dt, 4H), 6.535 (dt, 4H). ^{13}C NMR (100 MHz, $CDCl_3$), δ (ppm): 151.96, 147.17, 144.19, 144.03, 143.80, 143.61, 140.90, 140.35, 137.05, 135.86, 135.21, 132.09, 131.57, 131.52, 131.51, 130.74, 128.45, 127.70, 126.51, 126.45, 126.39, 126.35, 126.33, 126.22, 126.08, 125.95, 124.40, 123.08, 120.40. HRMS (MALDI-TOF): *m/z*: $[M]^+$ calcd for $C_{78}H_{55}N_4S$, 1078.4069, found 1078.4036.

Synthesis of BT-ATPE-1Br

To a mixture of ATPE (508 mg, 1.2 mmol), 4,7-dibromobenzo[c]-1,2,5-thiadiazole (1056 mg, 3.6 mmol), Cs_2CO_3 (1200 mg, 3.6 mmol), $Pd_2(dba)_3$ (110 mg, 0.12 mmol) and RuPhos (112mg, 0.24 mmol) under N_2, toluene (24 mL) was added. After 30 min of stirring in room temperature, the mixture was brought to reflux for 17 h. The reaction mixture was cooled to room temperature, water was added and extracted with DCM. The organic layers were combined, dried over anhydrous Na_2SO_4 and evaporated to dryness. The crude product was purified by silica gel column chromatography using DCM/hexane (v/v =1:4) as eluent to afford compound BT-ATPE-1Br as red solid (660 mg, 73.5%).

^1H NMR (400MHz, CD_2Cl_2) δ (ppm): 7.69 (d, 1H), 7.25 (t, 2H), 7.20-6.97 (m, 19H), 6.90 (d, 2H), 6.75 (d, 2H). ^{13}C NMR (100MHz, CD_2Cl_2) δ (ppm): 155.1, 151.1, 147.7, 146.3, 144.4, 144.3, 144.0, 141.5, 141.1, 140.1, 139.7, 133.0, 132.6, 131.8, 131.7, 129.7, 128.2, 127.0, 126.9, 124.7, 124.2, 123.9, 123.7, 107.8. HRMS (MALDI-TOF): *m/z:* $[M]^+$ calcd for $C_{38}H_{26}BrN_3S$, 635.1031, found 635.1066

Synthesis of BT-NATPE-1Br

Similar to the procedure for BT-ATPE-1Br, The crude product was purified by silica gel column chromatography using DCM/hexane (v/v =1:4) as eluent to afford compound BT-NATPE-1Br as red solid (772 mg, 75.0%).

^{1}H NMR (400MHz, CD_2Cl_2) δ (ppm): 7.89 (t, 2H), 7.79 (d, 1H), 7.58 (d, 1H), 7.48 (t, 1H), 7.42 (t, 1H), 7.36 (t, 1H), 7.25-7.03 (m, 16H), 6.88 (d, 2H), 6.81 (d, 1H), 6.66 (d, 2H). ^{13}C NMR (100MHz, CD_2Cl_2) δ (ppm): 155.1, 150.5, 147.1, 144.5, 144.3, 144.1, 143.7, 141.4, 141.1, 140.8, 138.9, 135.7, 133.0, 132.4, 131.9, 131.8, 130.8, 129.0, 128.2, 127.3, 127.0, 127.0, 126.9, 126.8, 126.5, 124.3, 122.2, 121.8, 107.1. HRMS (MALDI-TOF): *m/z:* $[M]^+$ calcd for $C_{42}H_{28}BrN_3S$, 685.1187, found 685.1153

Synthesis of BT-NATPE-BA

Under N_2, a mixture of BT-NATPE-1Br (660 mg, 0.874 mmol), 4-Formylphenylboronic acid (158 mg, 1.049 mmol), K_2CO_3 (482 mg, 3.495 mmol) and $Pd(PPh_3)_4$ (40.5 mg, 0.035 mmol), water purged with N_2 (8.7mL) and THF (29.1mL) were added. The reaction mixture was refluxed overnight. The reaction mixture was cooled to room temperature, washed with water and then extracted using DCM. The organic layers were combined, dried over anhydrous Na_2SO_4 and evaporated to dryness. The crude product was purified by silica gel column chromatography using DCM/hexane (v/v =1:4) as eluent to afford compound BT-NATPE-BA as orange solid (600 mg, 96.2%).

^{1}H NMR (400MHz, $CDCl_3$) δ (ppm): 10.08 (s, 1H), 8.09 (d, 2H), 8.00 (d, 2H), 7.91 (d, 2H), 7.80(d, 1H), 7.75 (d, 1H), 7.51-7.43 (m, 2H), 7.37-7.29 (m, 2H), 7.19-7.02 (m, 15H), 6.97 (d, 1H), 6.90 (d, 2H), 6.72 (d, 2H). ^{13}C NMR (100MHz, $CDCl_3$) δ (ppm): 192.1, 154.7, 150.6, 146.8, 144.2, 143.9, 143.7, 143.7, 143.5, 140.9, 140.9, 140.8, 138.6, 135.5, 132.3, 131.7, 131.6, 131.5, 130.7, 130.1, 129.7, 129.5, 128.6, 127.9, 127.8, 127.1, 126.7, 126.6, 126.5, 126.4, 126.2, 124.1, 122.2, 120.4. HRMS (MALDI-TOF): *m/z:* $[M]^+$ calcd for $C_{49}H_{33}N_3OS$, 735.2344, found 735.2340

Synthesis of BT-ATPE-Ph

Under N_2, a mixture of BT-ATPE-Br (90 mg, 0.141 mmol), phenylboronic acid (19 mg, 0.156 mmol), K_2CO_3 (78 mg, 0.567 mmol) and $Pd(PPh_3)_4$ (7 mg, 0.006 mmol), water purged with N_2 (1.4mL) and THF (4.7mL) were added. The reaction mixture was brought to reflux overnight. The reaction mixture was cooled to room temperature, added with water and extracted with DCM. The organic layers were combined, dried over anhydrous Na_2SO_4 and evaporated to dryness. The crude product was purified by silica gel column chromatography using DCM/hexane (v/v =1:4 to v/v = 1:2) as eluent to afford compound BT-ATPE-Ph as orange solid (89 mg, 99.2%).

^1H NMR (400MHz, CD_2Cl_2) δ (ppm): 7.84 (d, 2H), 7.53 (d, 1H), 7.44 (t, 2H), 7.34 (t, 1H), 7.20-6.97 (m, 21H), 6.85 (d, 2H), 6.73 (d, 2H). ^{13}C NMR (100MHz, CD_2Cl_2) δ (ppm): 155.5, 152.1, 148.0, 146.6, 144.5, 144.4, 144.2, 141.4, 141.2, 139.4, 139.3, 138.2, 132.6, 131.9, 131.8, 130.1, 129.7, 129.1, 129.0, 128.4, 128.2, 127.0, 126.9, 124.5, 123.9, 123.5. HRMS (MALDI-TOF): *m/z:* $[M]^+$ calcd for $C_{44}H_{31}N_3S$, 633.2239, found 633.2266

Synthesis of BT-ATPE-Py

Under N_2, a mixture of BT-ATPE-1Br (90 mg, 0.141 mmol), 4-pyridinylboronic acid (20 mg, 0.156 mmol), K_2CO_3 (78 mg, 0.567 mmol) and $Pd(PPh_3)_4$ (7 mg, 0.006 mmol), water purged with N_2 (1.4mL) and THF (4.7mL) were added. The reaction mixture was refluxed overnight. The reaction mixture was cooled to room temperature and extracted using DCM against water. The organic layers were combined, dried over anhydrous Na_2SO_4 and evaporated to dryness. The crude product was purified by silica gel column chromatography using DCM/hexane (v/v =1:4 to v/v = 1:2) as eluent to afford compound BT-ATPE-Py as red solid (80 mg, 87.9%).

^1H NMR (400MHz, CD_2Cl_2) δ (ppm): 8.61 (d, 2H), 7.82 (d, 2H), 7.64 (d, 1H), 7.20 (t, 2H), 7.13-6.96 (m, 19H), 6.86 (d, 2H), 6.73 (d, 2H). ^{13}C NMR (100MHz, CD_2Cl_2) δ (ppm): 155.0, 151.7, 150.5, 147.8, 146.3, 145.2, 144.5, 144.3, 144.1, 141.6, 141.1, 139.9, 132.6, 132.5, 132.4, 131.9, 131.8, 130.0, 129.8, 129.1, 129.0, 128.2, 127.0, 126.9, 126.0, 125.0, 124.4, 124.0, 123.7, 122.9. HRMS (MALDI-TOF): *m/z:* $[M]^+$ calcd for $C_{43}H_{30}N_4S$, 634.2191, found 634.2179

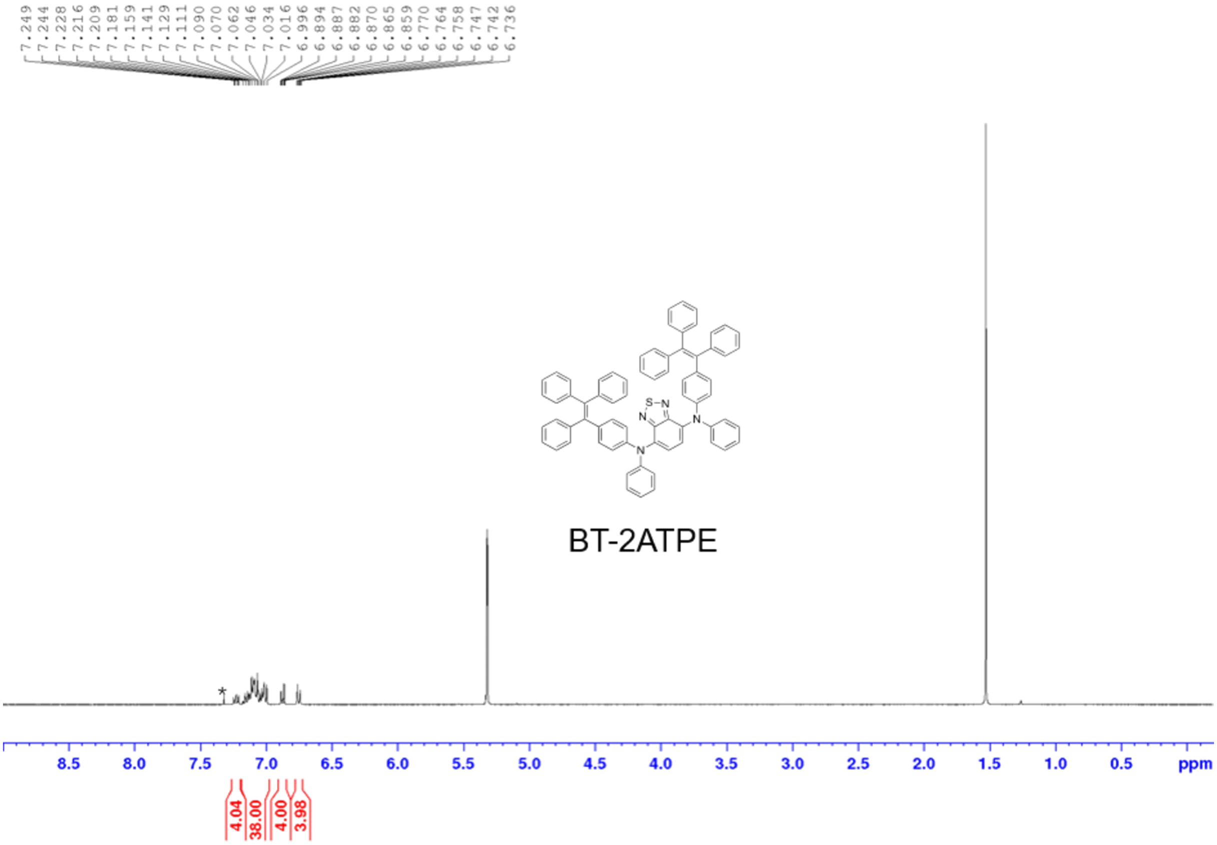

Figure S1. ^{1}H NMR spectrum of BT-2ATPE in CD_2Cl_2

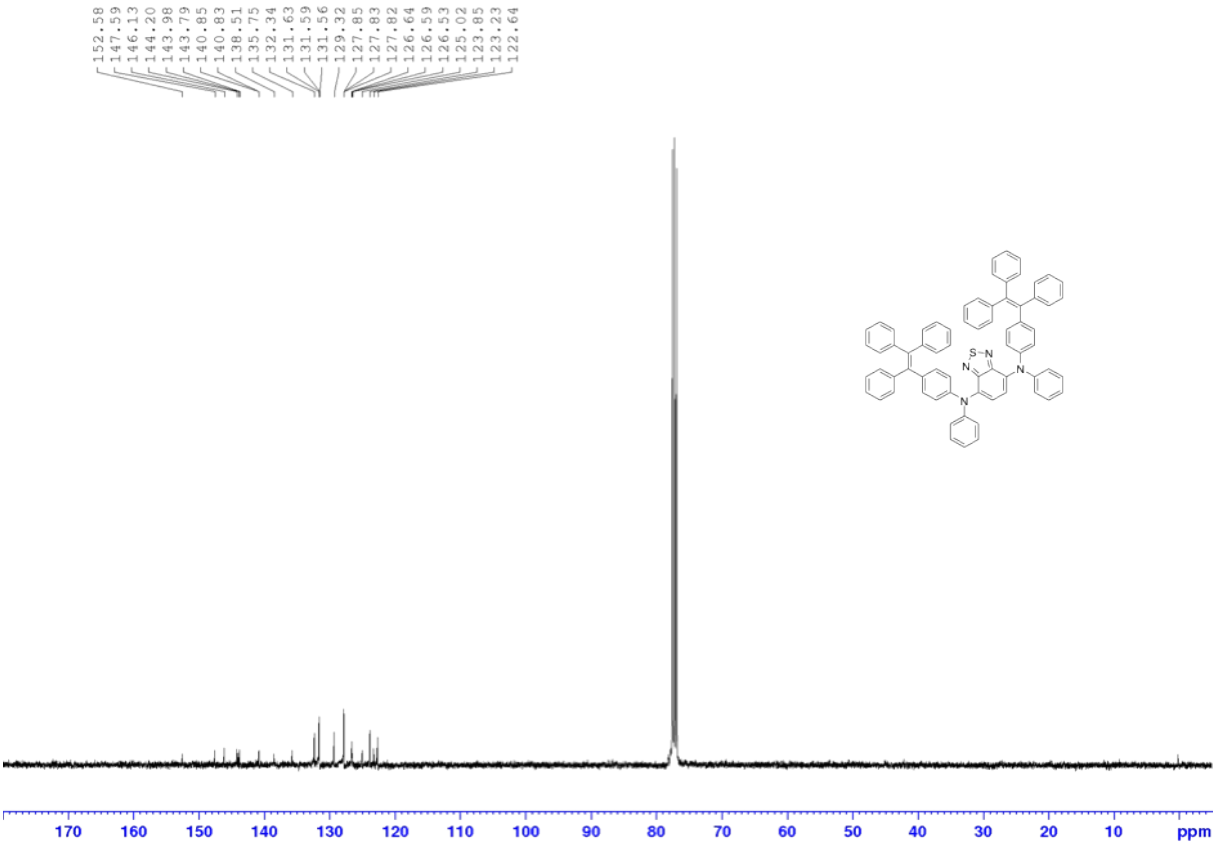

Figure S2.^{13}C NMR spectrum of BT-2ATPE in $CDCl_3$

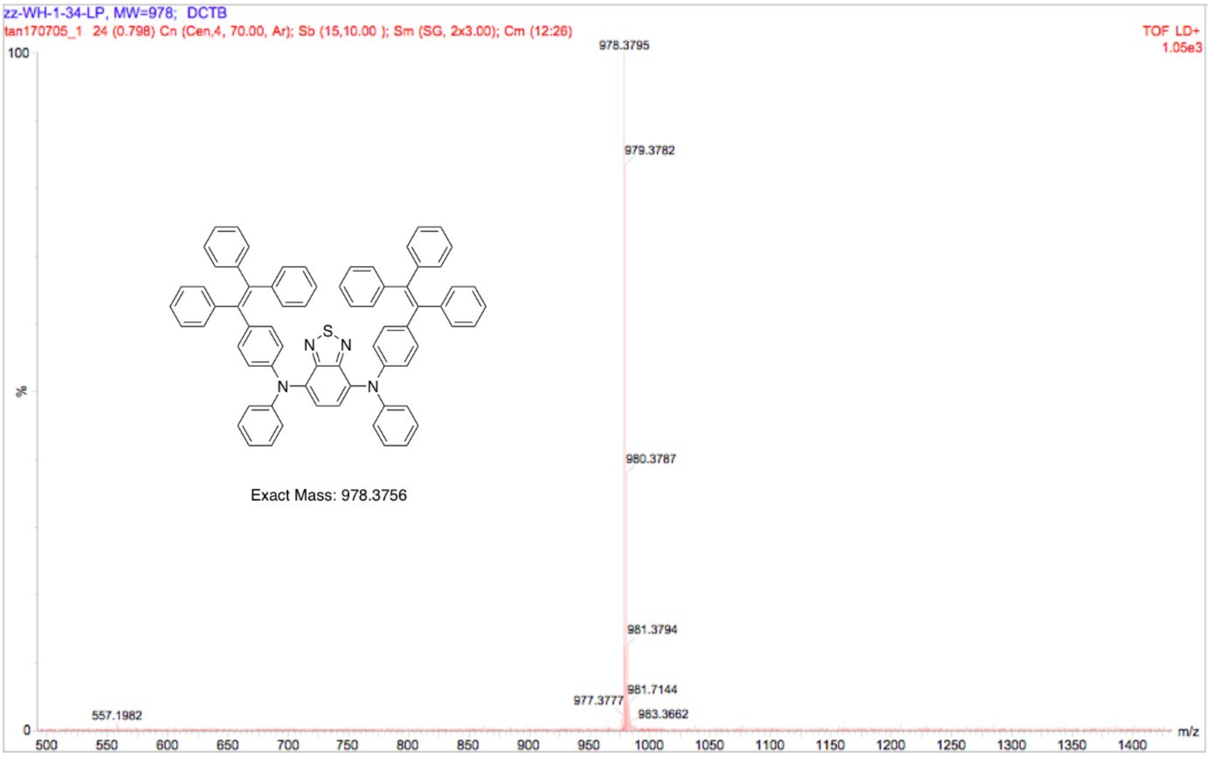

Figure S3.High resolution mass spectrum of BT-2ATPE

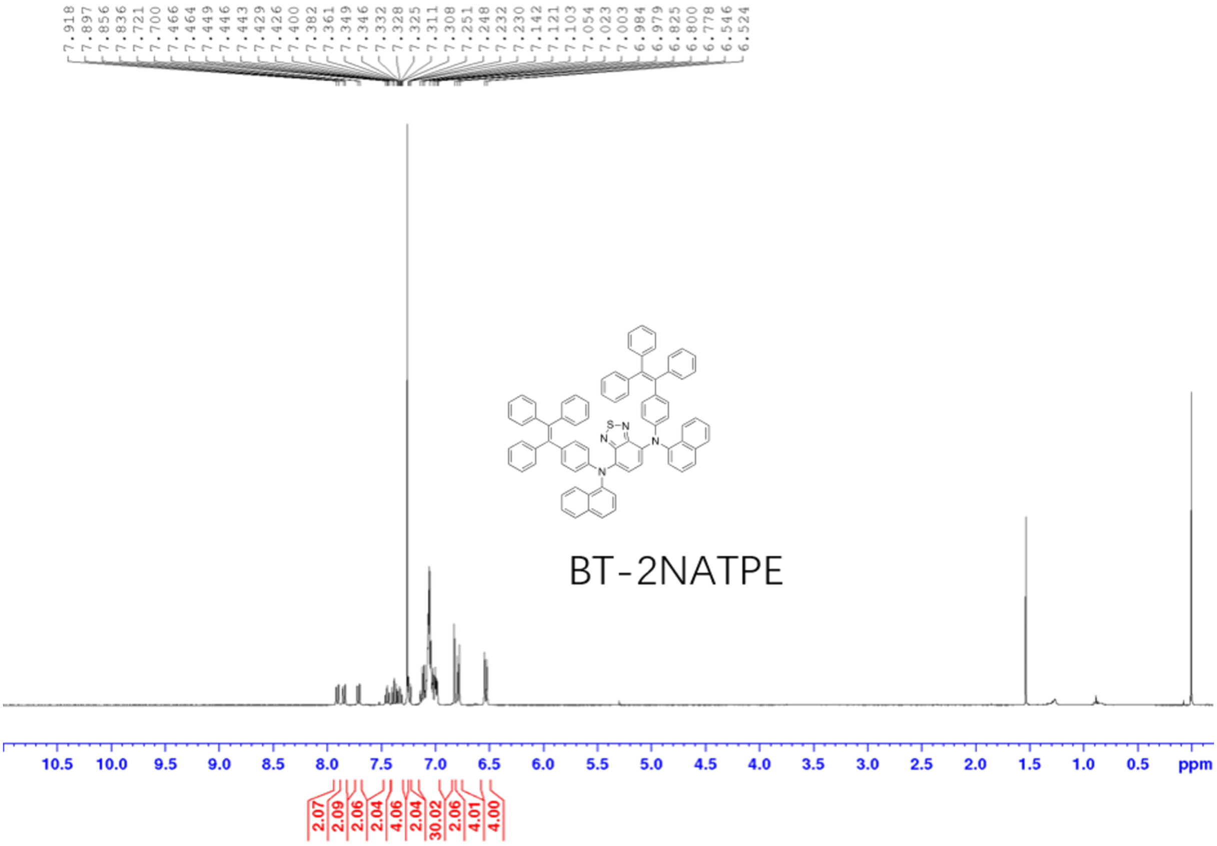

Figure S4.^{1}H NMR spectrum of BT-2NATPE in $CDCl_3$

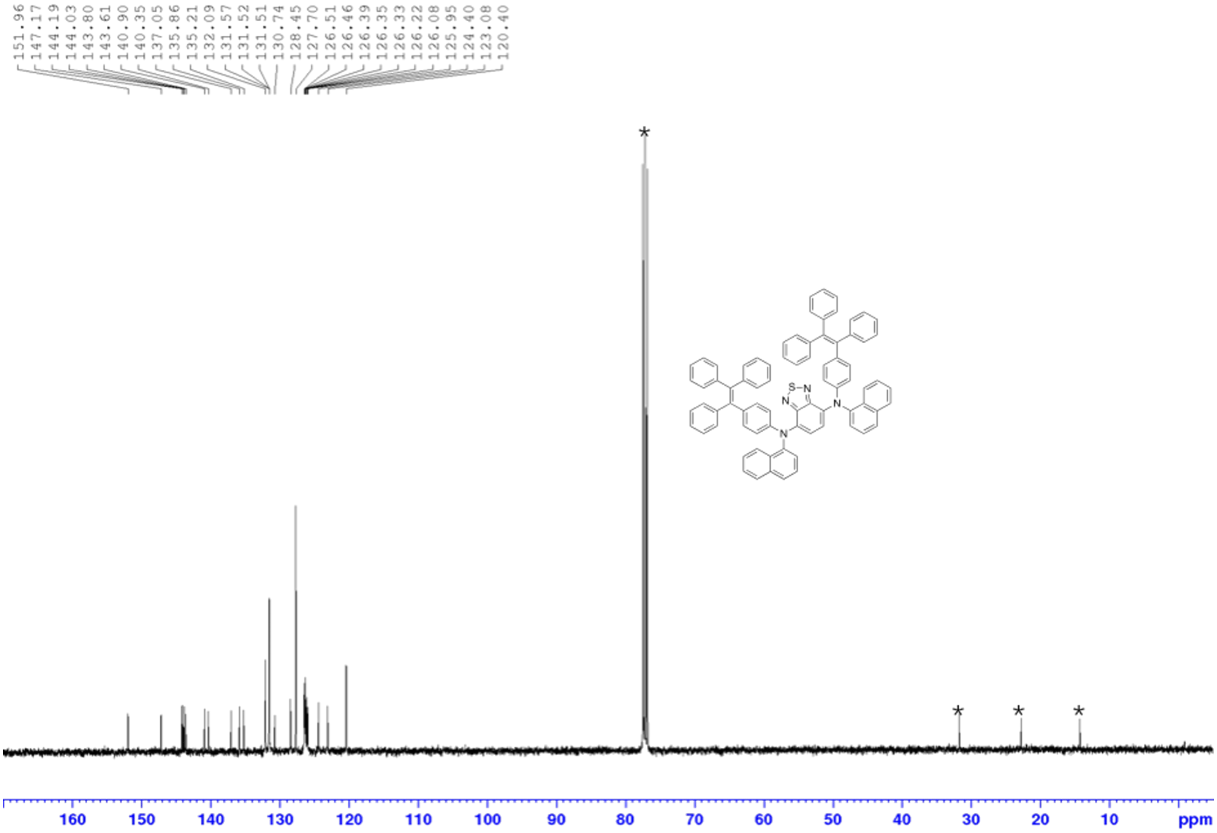

Figure S5.^{13}C NMR spectrum of BT-2NATPE in $CDCl_3$

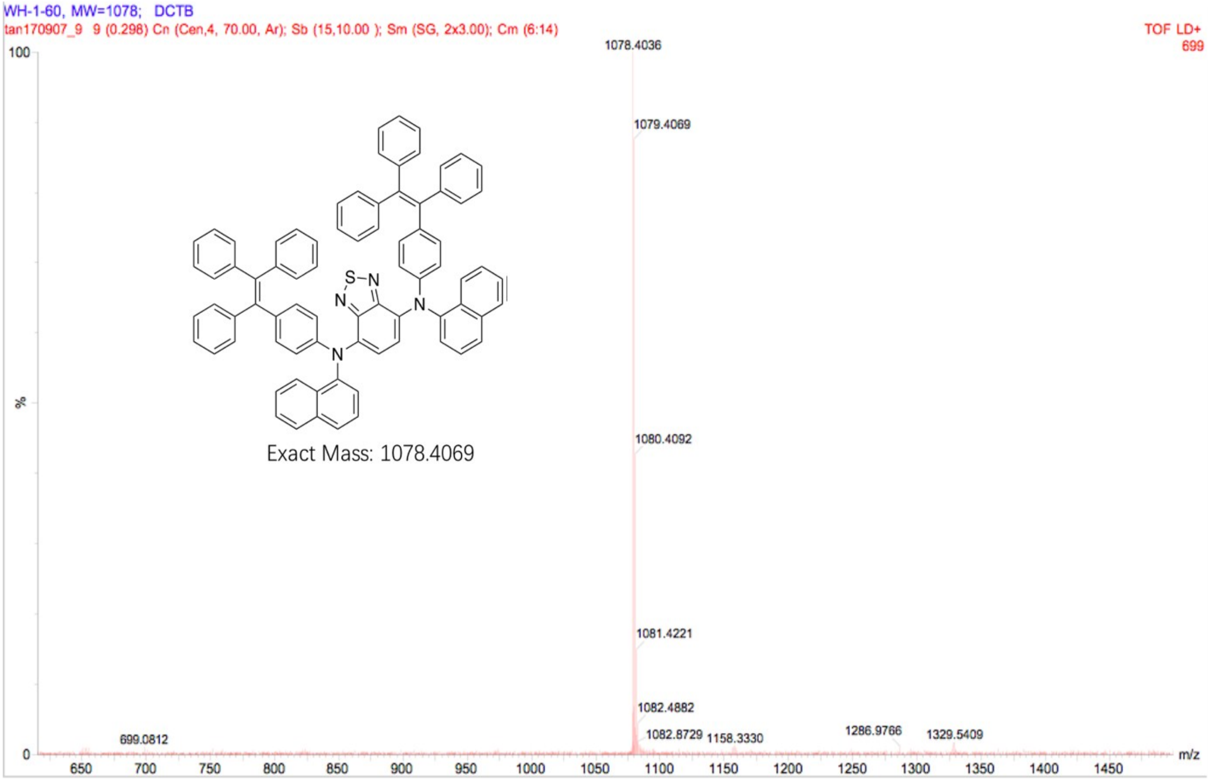

Figure S6. High resolution mass spectrum of BT-2NATPE

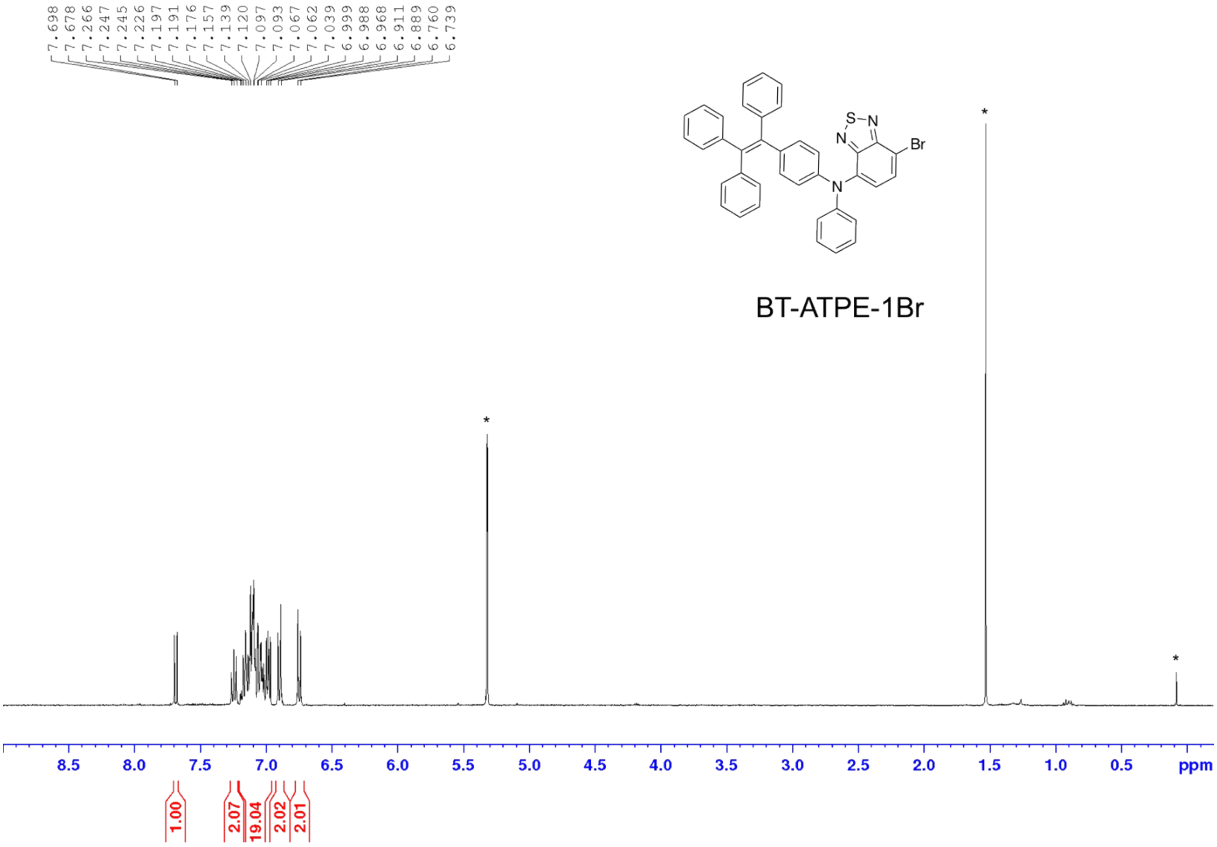

Figure S7.^{1}H NMR spectrum of BT-2ATPE-1Br in CD_2Cl_2

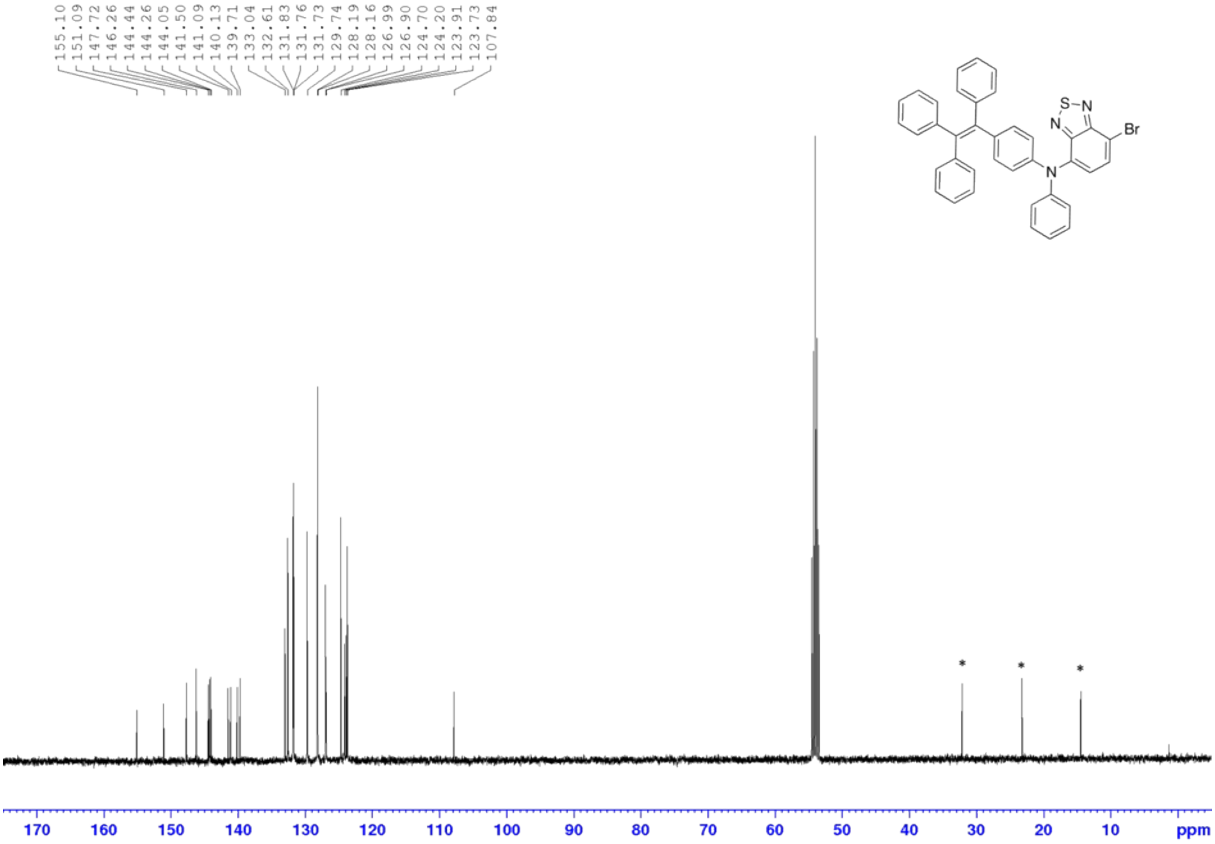

Figure S8.^{13}C NMR spectrum of BT-2ATPE-1Br in CD_2Cl_2

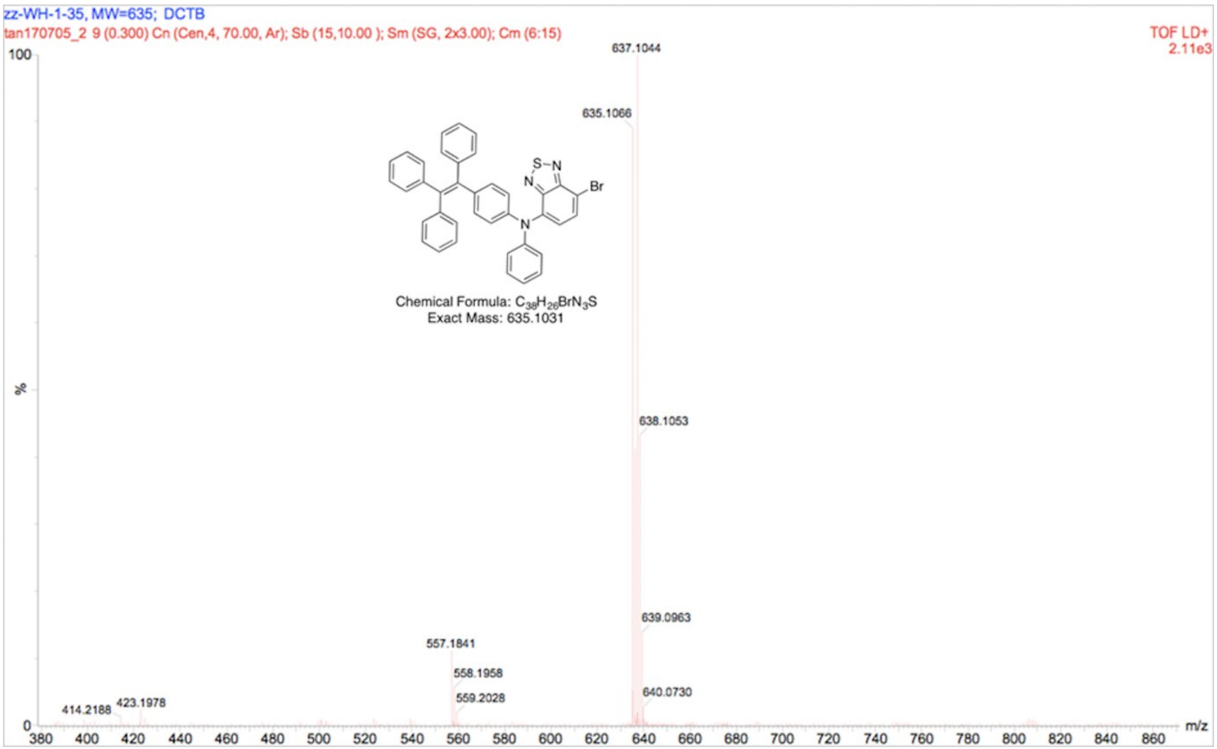

Figure S9.High resolution mass spectrum of BT-2ATPE-1Br.

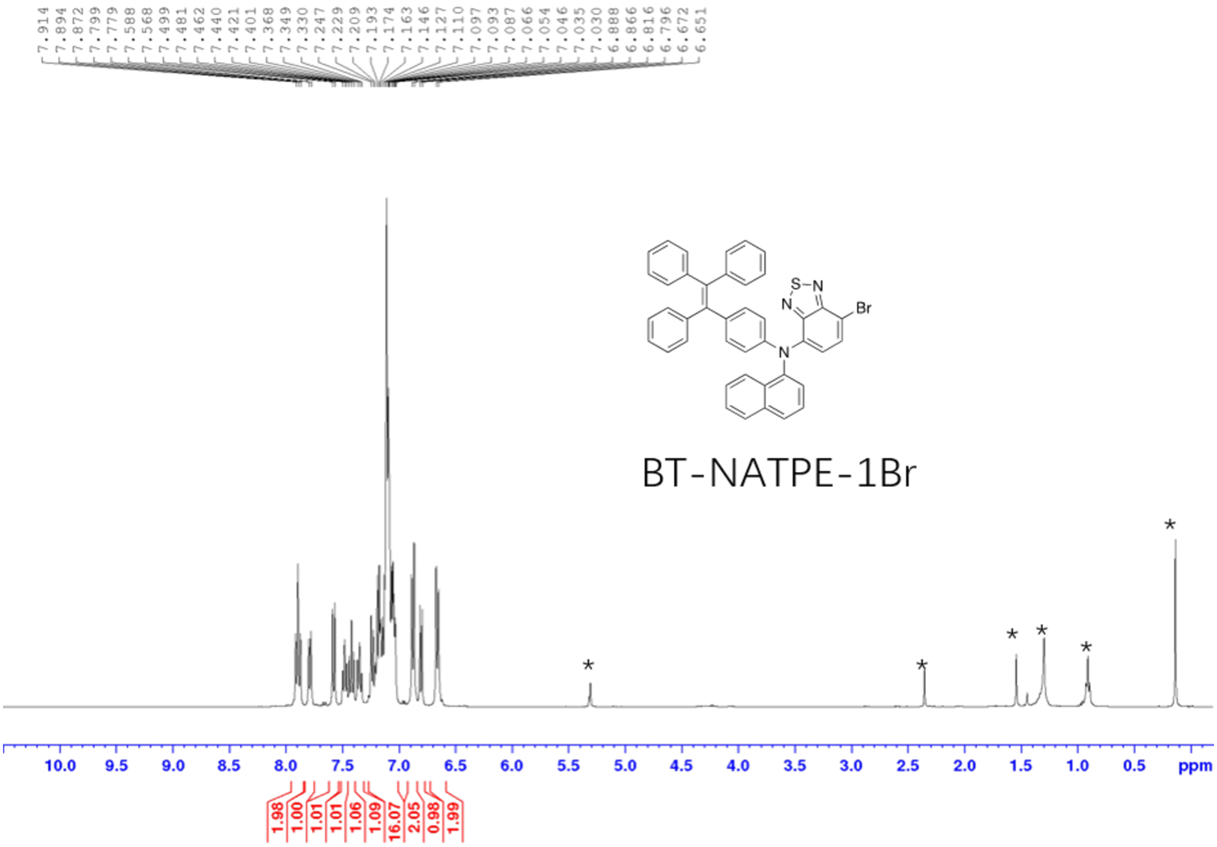

Figure S10.^{1}H NMR spectrum of BT-NATPE-1Br in CD_2Cl_2

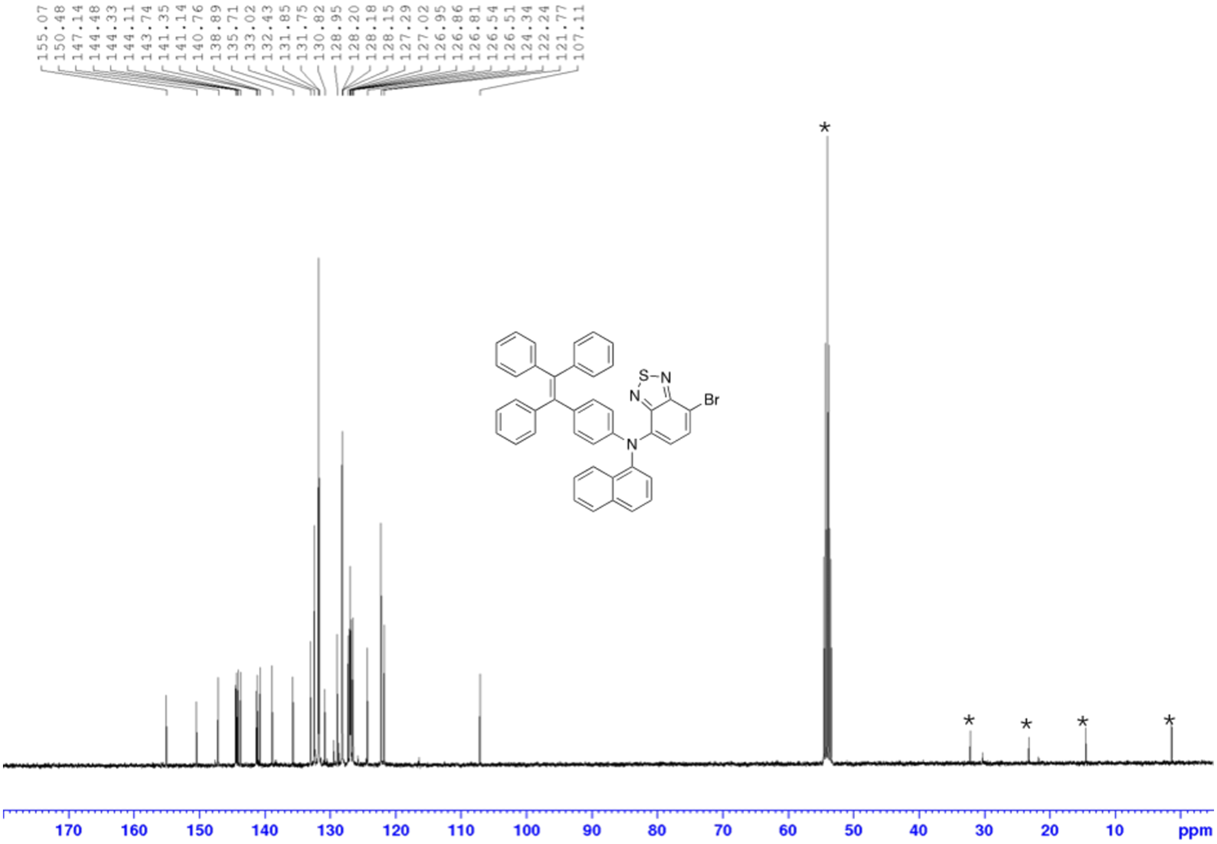

Figure S11.^{13}C NMR spectrum of BT-NATPE-1Br in CD_2Cl_2

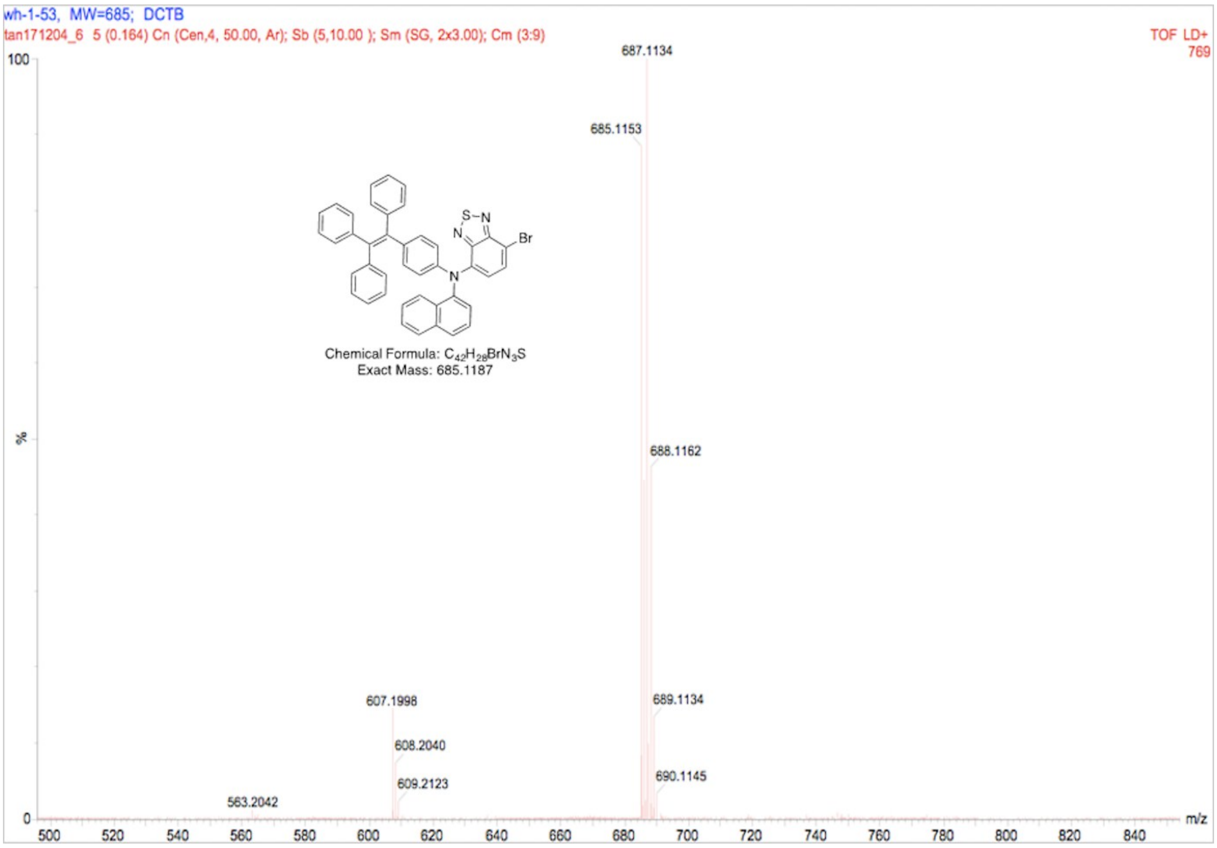

Figure S12. High resolution mass spectrum of BT-NATPE-1Br

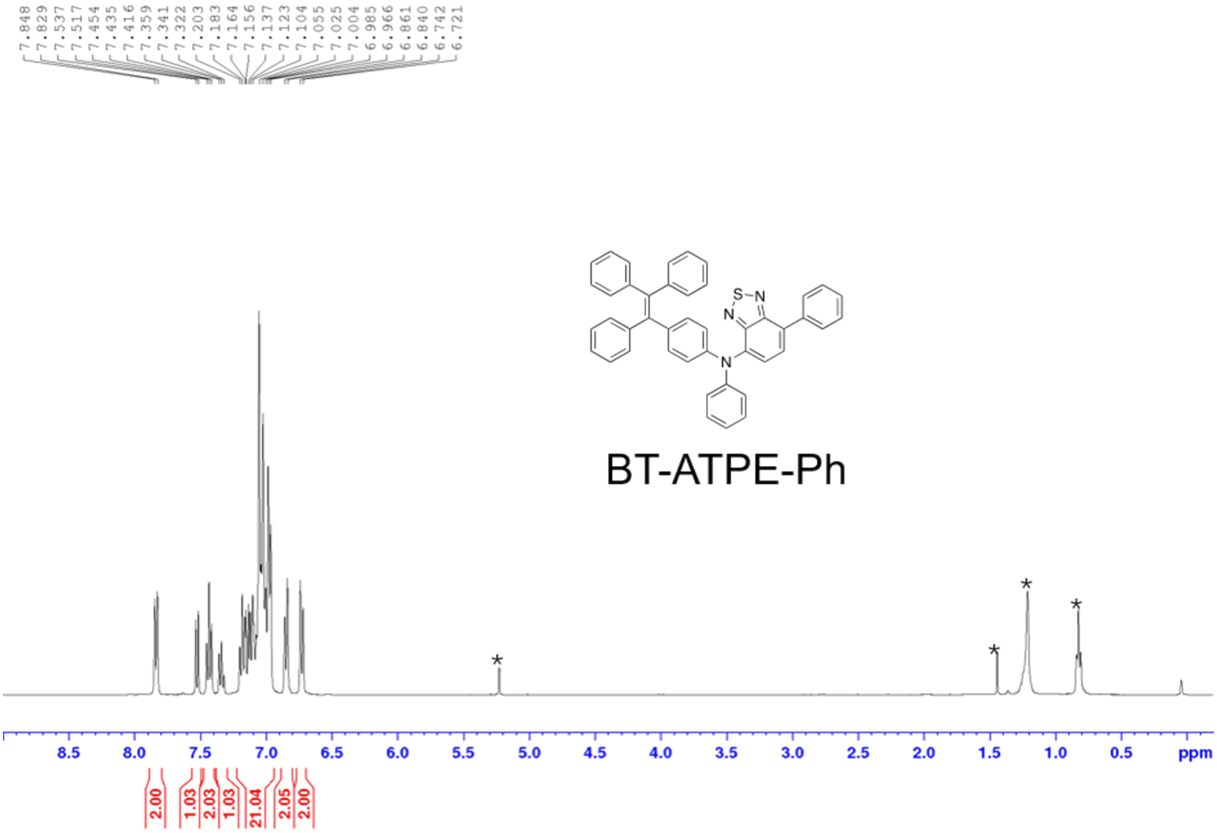

Figure S13. ^{1}H NMR spectrum of BT-ATPE-Ph in CD_2Cl_2

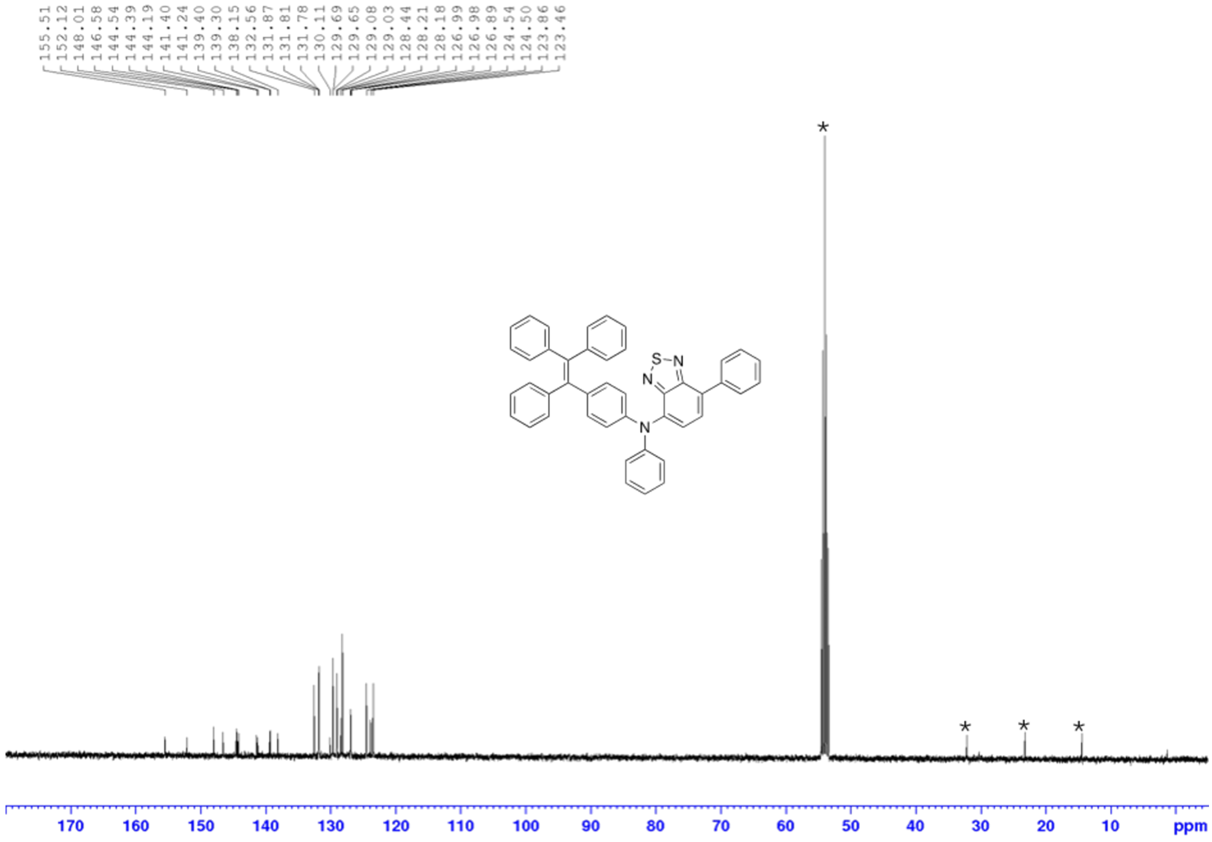

Figure S14. ^{13}C NMR spectrum of BT-ATPE-Ph in CD_2Cl_2

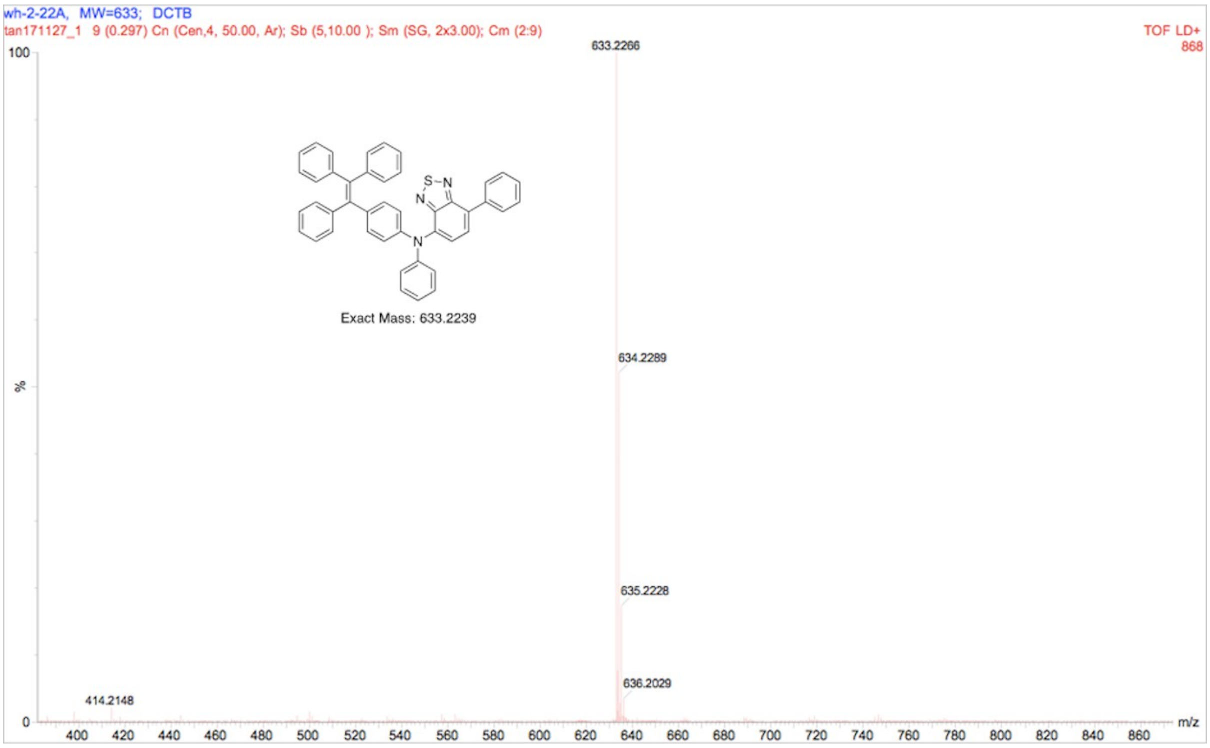

Figure S15. High resolution mass spectrum of BT-ATPE-Ph

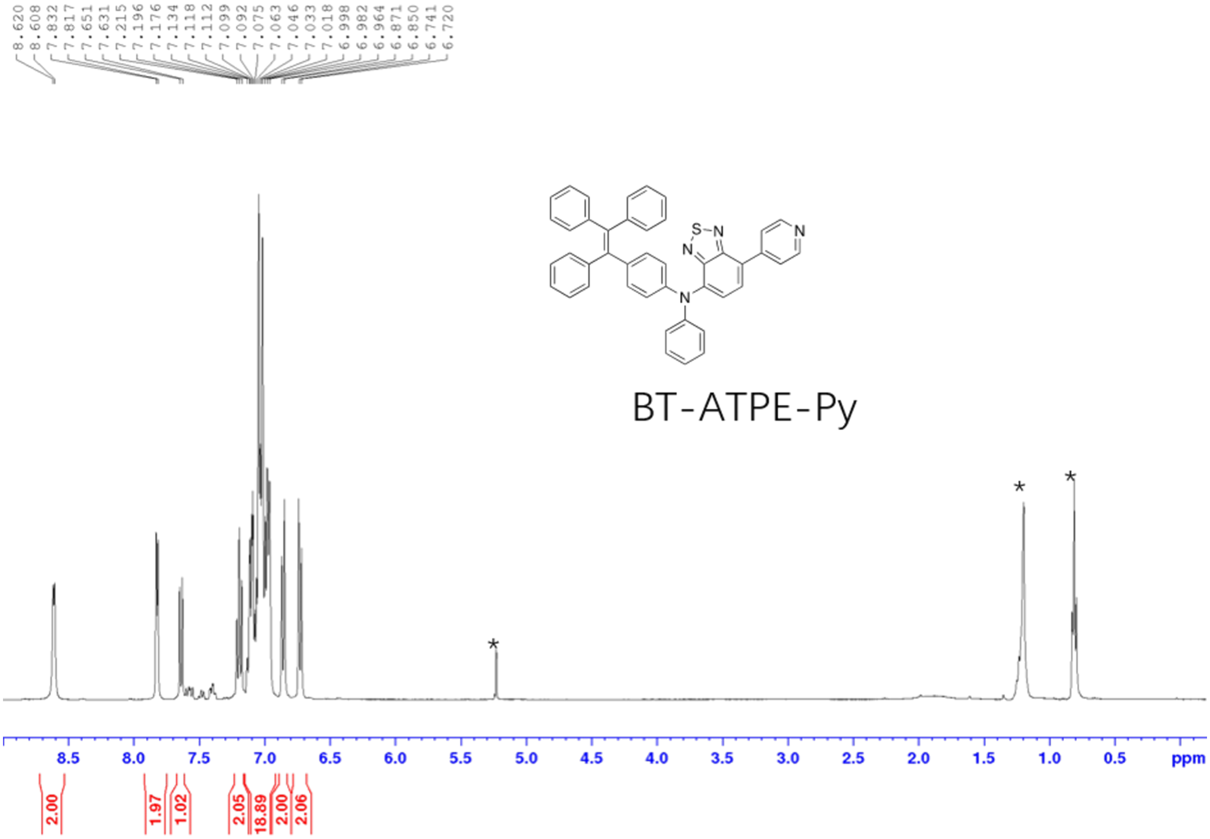

Figure S16. ^{1}H NMR spectrum of BT-ATPE-Py in CD_2Cl_2

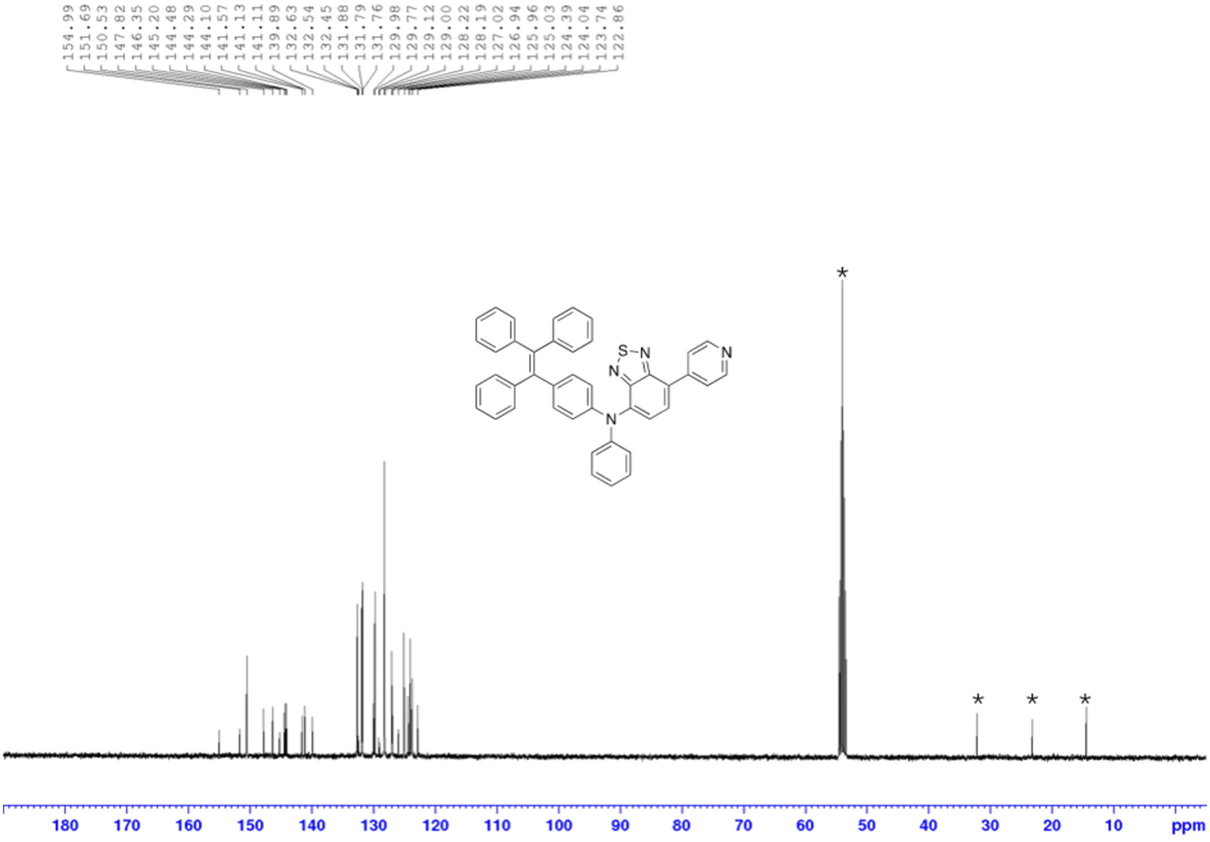

Figure S17. ^{13}C NMR spectrum of BT-ATPE-Py in CD_2Cl_2

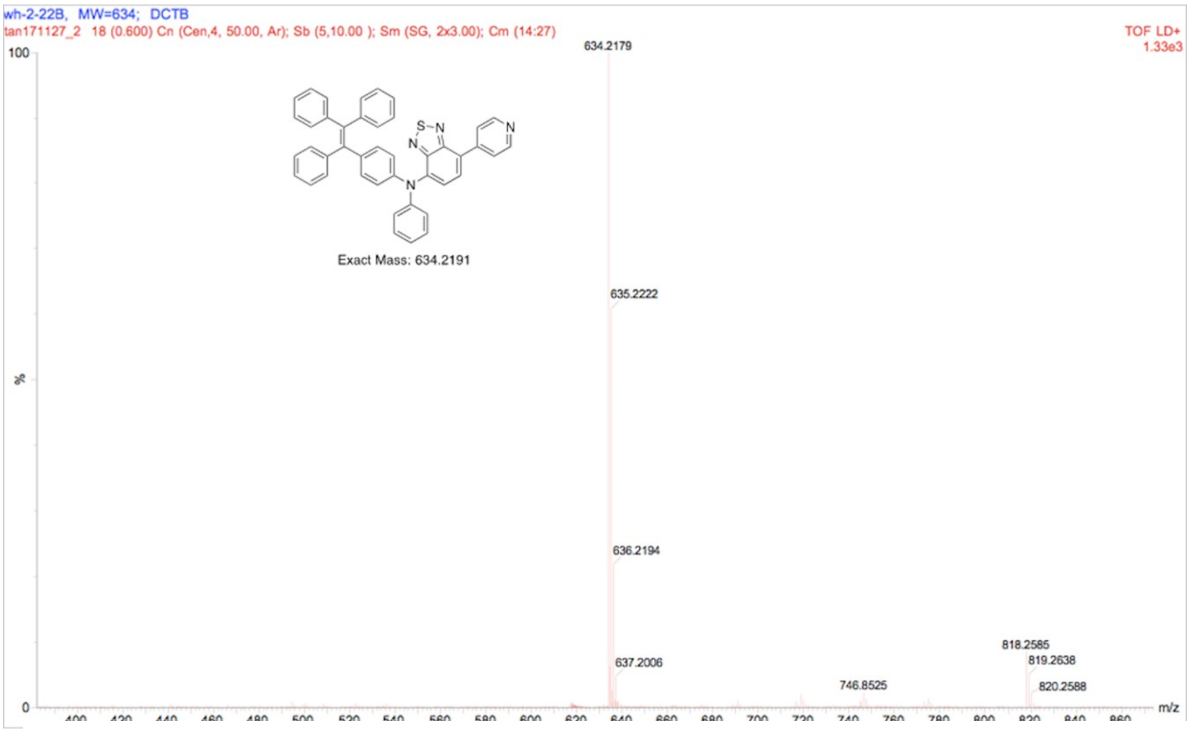

Figure S18. High resolution mass spectrum of BT-ATPE-Py

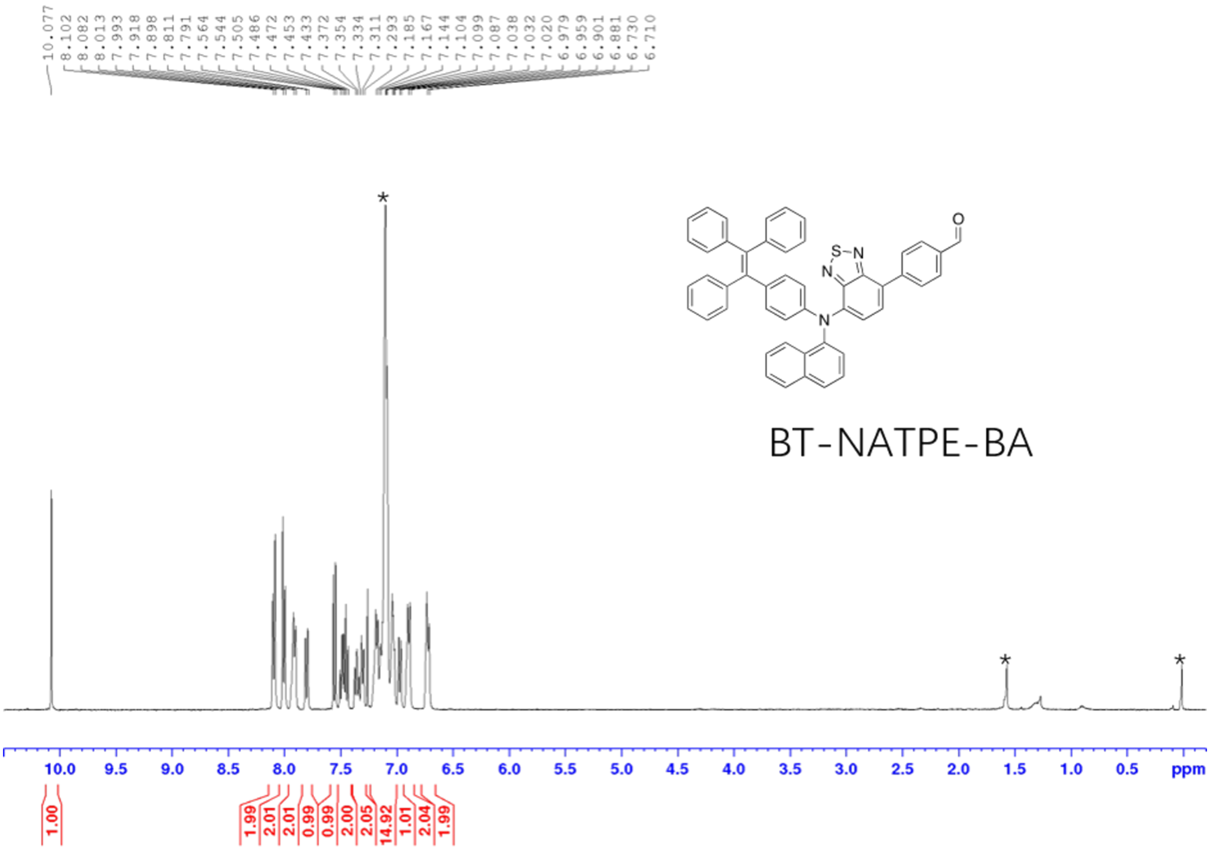

Figure S19.^{1}H NMR spectrum of BT-NATPE-BA in $CDCl_3$

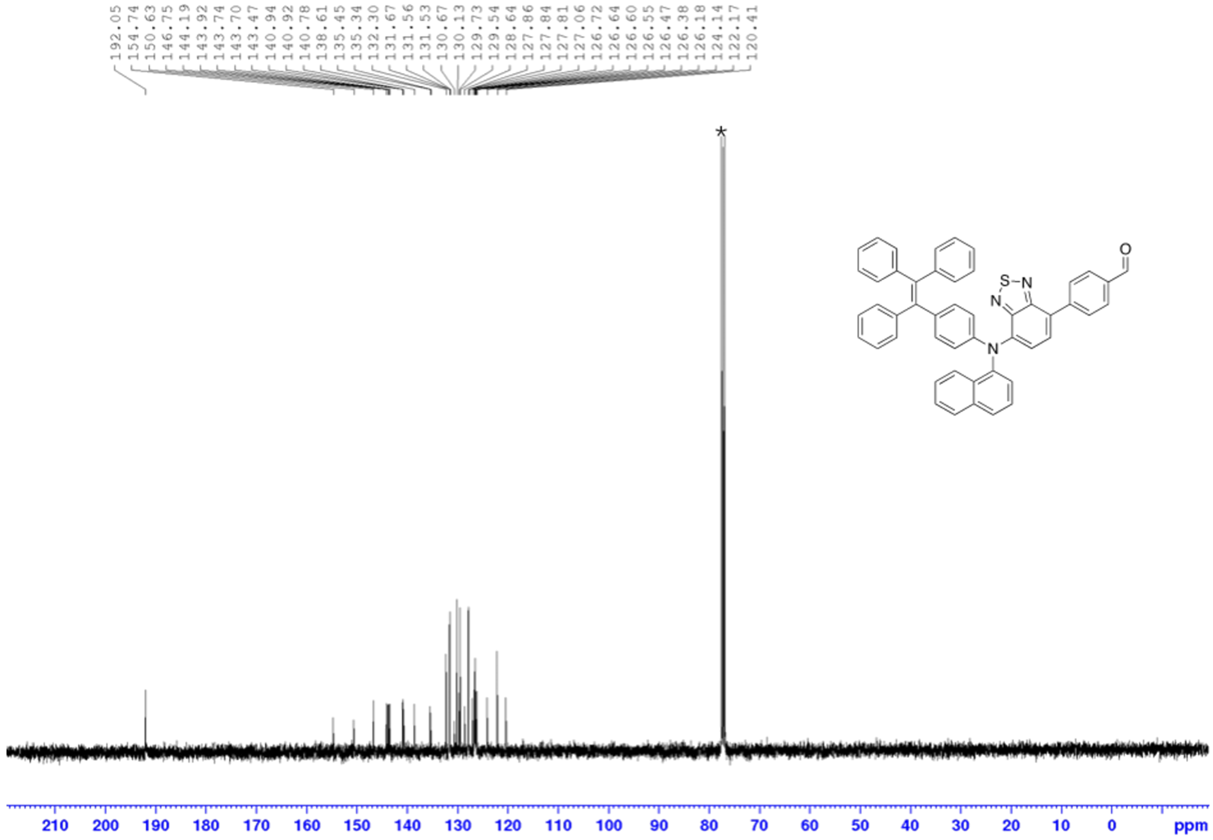

Figure S20.^{13}C NMR spectrum of BT-NATPE-BA in $CDCl_3$

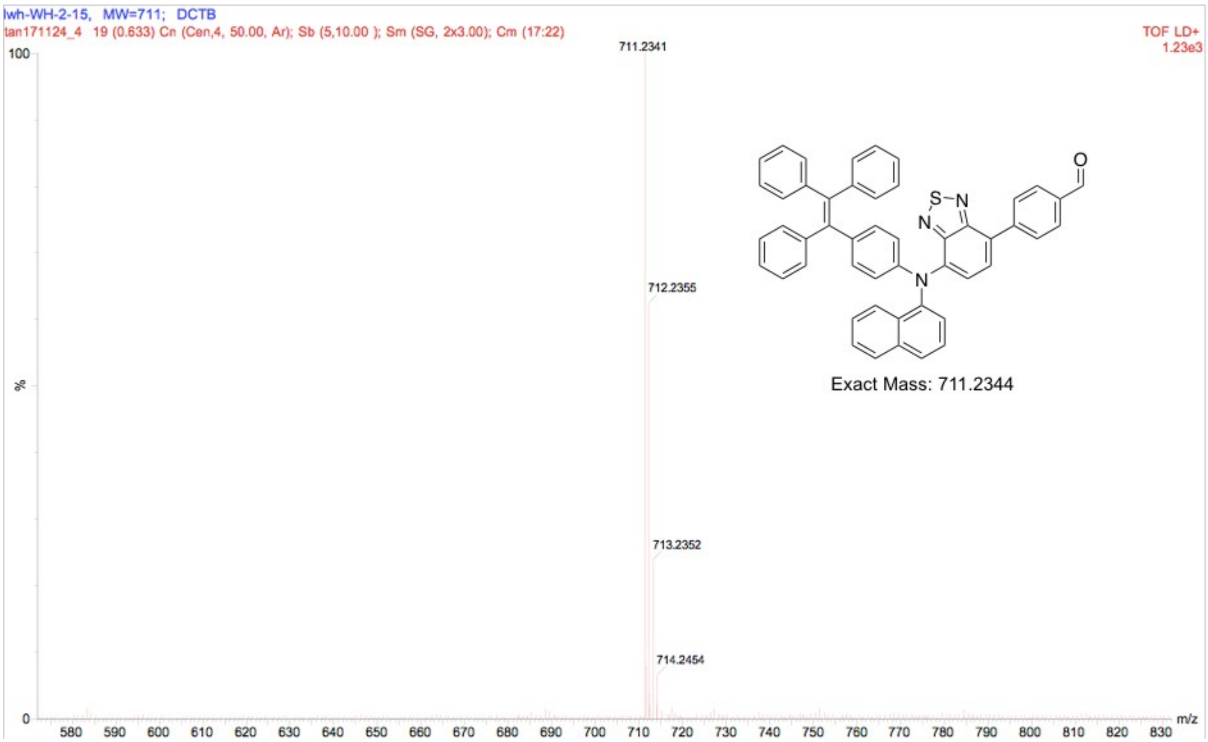

Figure S21. High resolution mass spectrum of BT-NATPE-BA

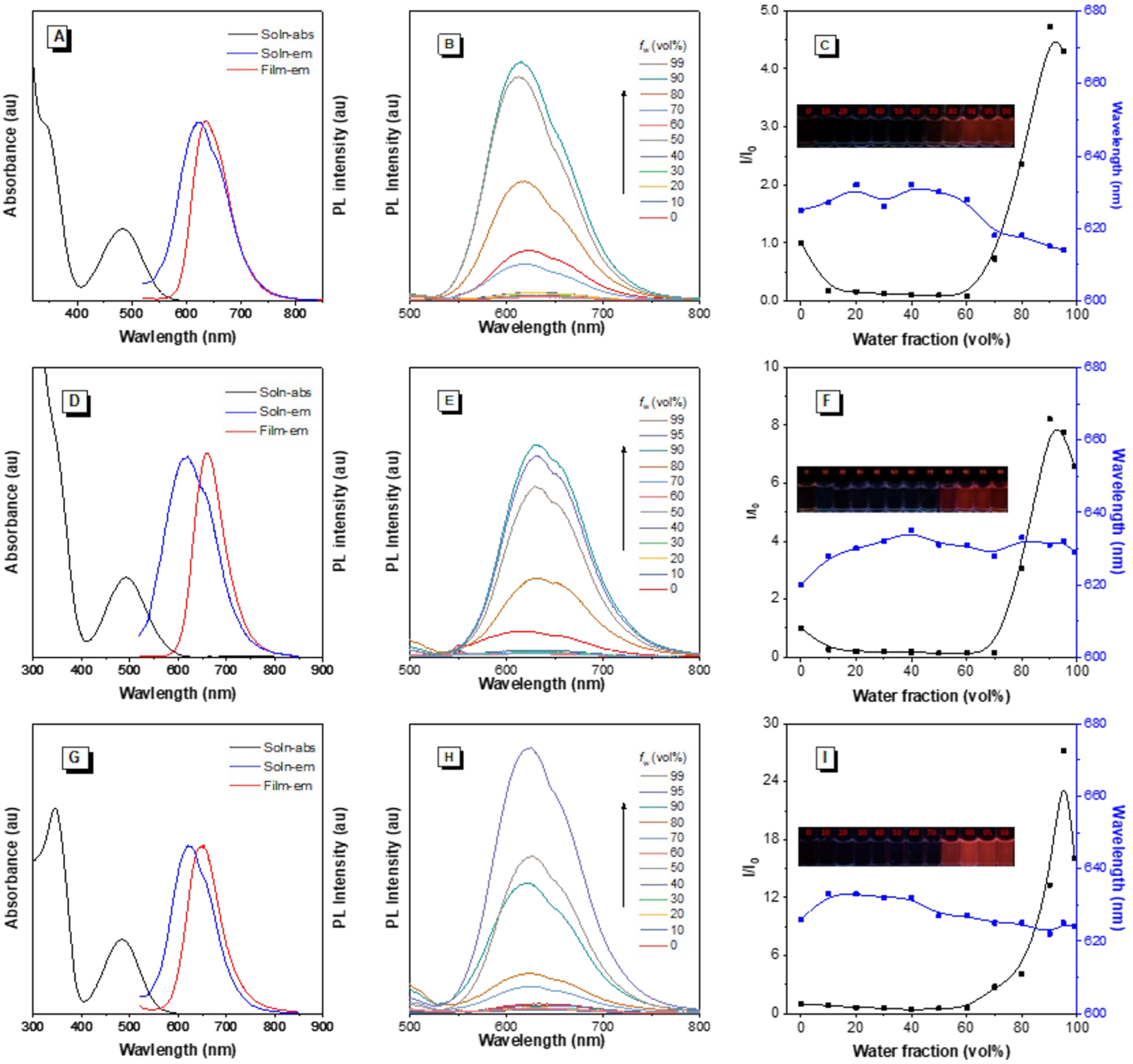

Figure S22. (A, D, G) UV and PL spectra of (A) BT-ATPE-Ph, (D) BT-ATPE-Py and (G) BT-NATPE-BA in dilute THF solutions (10 µM) and thin films. (B, E, H) PL spectra of (B) BT-ATPE-Ph, (E) BT-ATPE-Py and (H) BT-NATPE-BA in THF/H_2O mixtures with different water fractions (f_w). Concentration: 10 µM The absorption maximum of each compound was chosen as its excitation wavelength; (C, F, I). The plots of the emission maximum and the relative emission intensity (I/I_0) versus the composition of the aqueous mixture of (C) BT-ATPE-Ph, (F) BT-ATPE-Py and (I) BT-NATPE-BA, I_0 = PL intensity in pure THF. Inset: Fluorescence photographs of BT-ATPE-Ph, BT-ATPE-Py and BT-NATPE-BA in THF/H_2O mixtures with different water fractions (f_w) taken under 365 nm UV irradiation, Concentration: 10 µM. The absorption maximum of each compound was chosen as its excitation wavelength

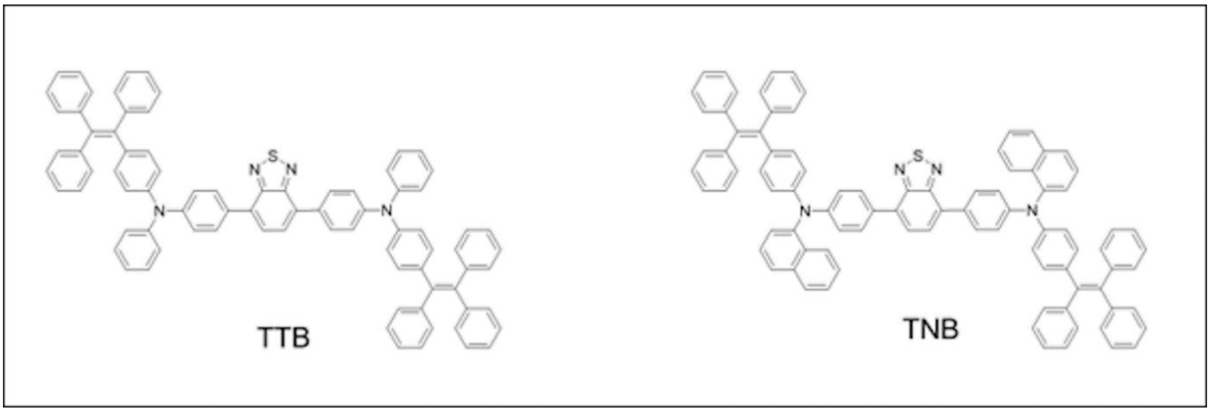

Figure S23. Molecular structures of TTB and TNB

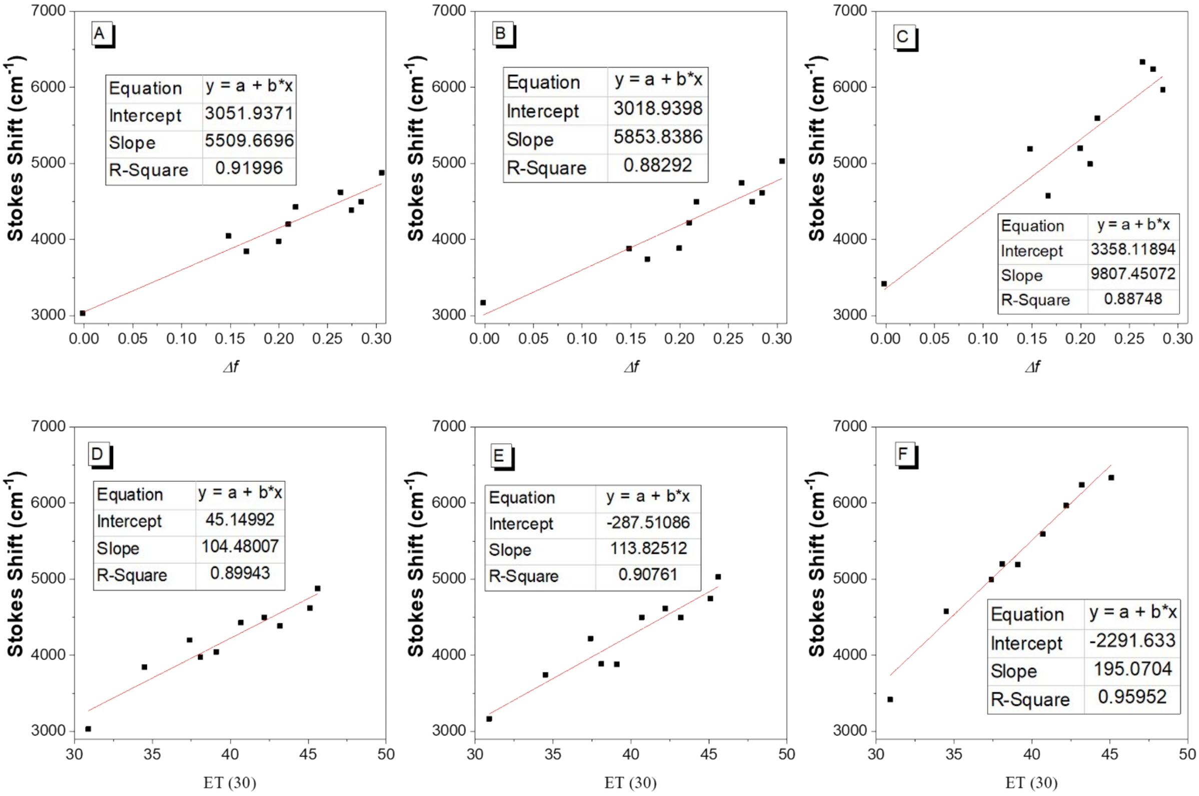

Figure S24 (A-C) Lippert-Mataga plots of (A) BT-2ATPE, (B) BT-2NATPE and (C) TTB (D-F) Plots of Stokes shifts against E_T (30) of different solvents for (D) BT-2ATPE, (E) BT-2NATPE and (F) TTB.

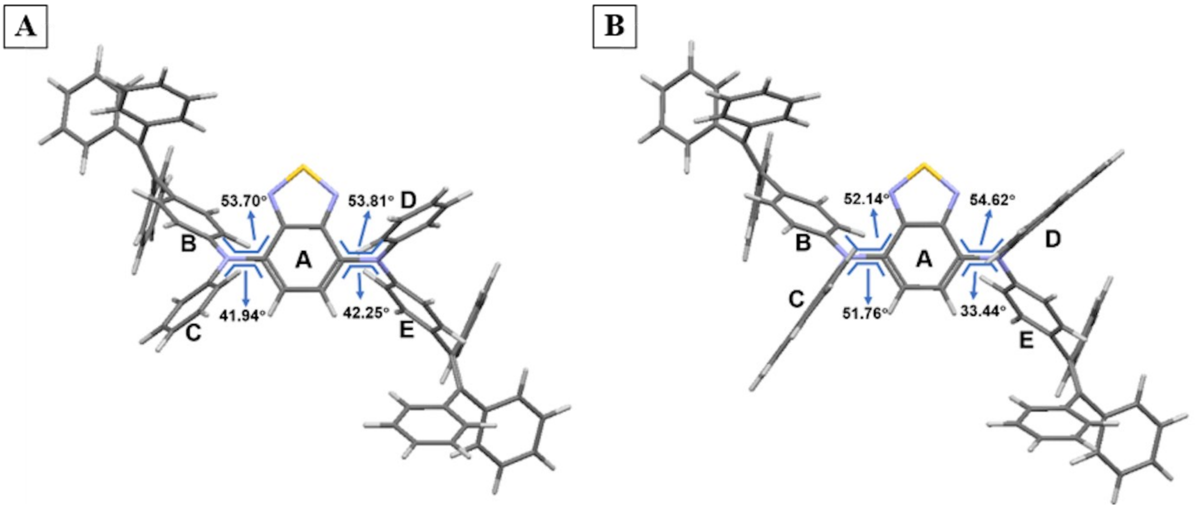

Figure S25. Optimized geometric structures in the gas phase of (A) BT-2ATPE and (B) BT-2NATPE calculated at B3LYP/6-31G(d,p) level.

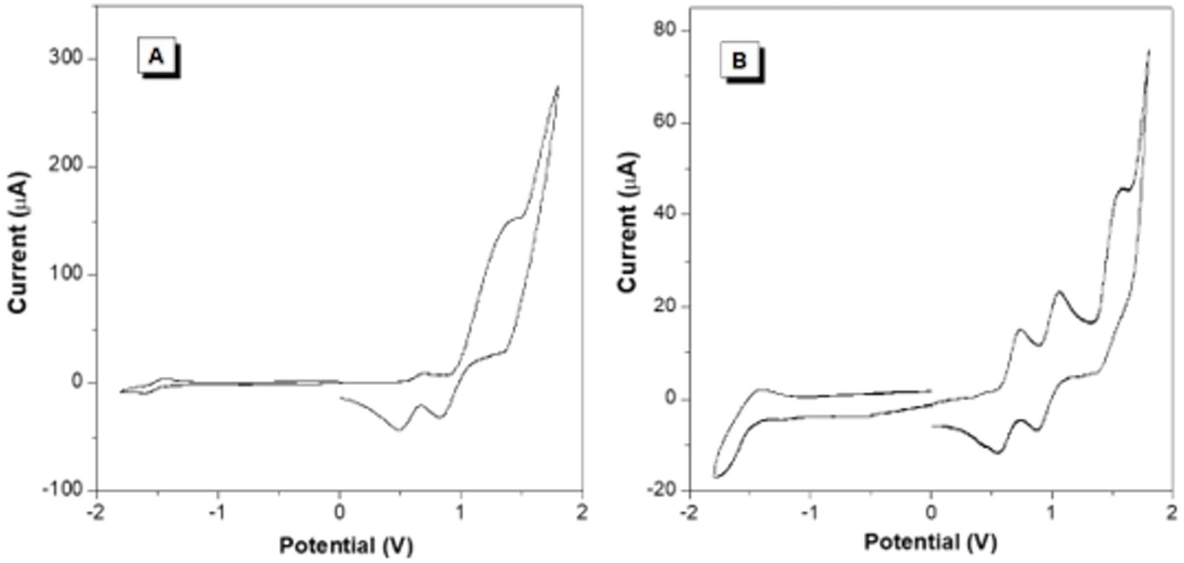

Figure S26. Cyclic voltammetry curves of (A) BT-2ATPE and (B) BT-2NATPE with 0.1 M $BuN_4^+PF_6^-$ in CH_2Cl_2 solution at a scan rate of 100 mV/s

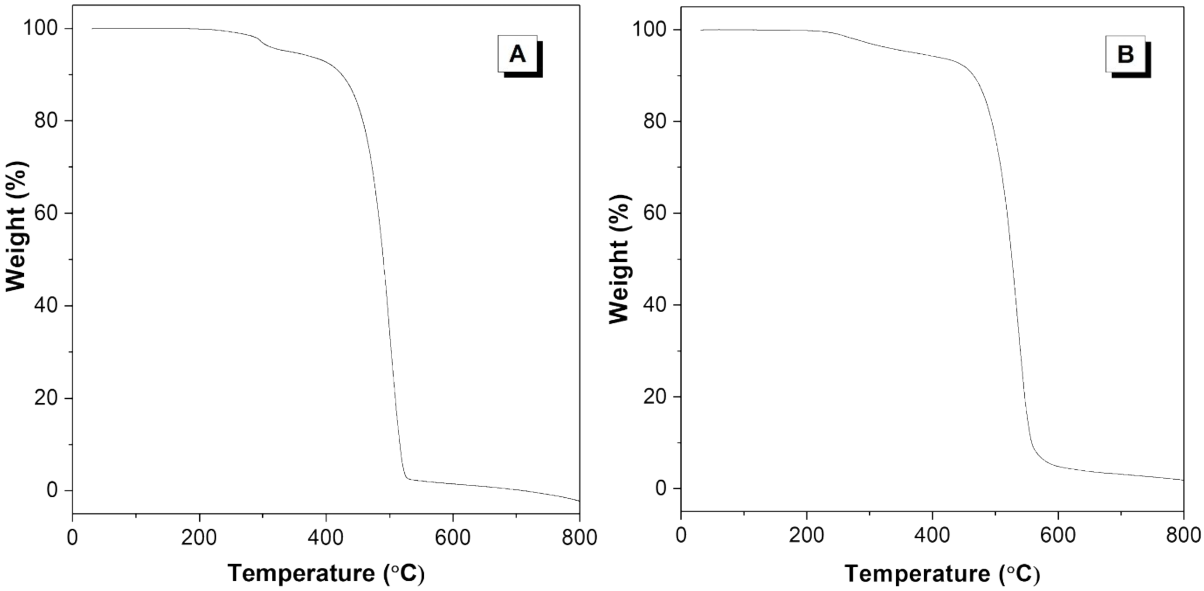

Figure S27. TGA curves of (A) BT-2ATPE and (B) BT-2NATPE recorded under nitrogen at a heating rate of 10 °C/min.

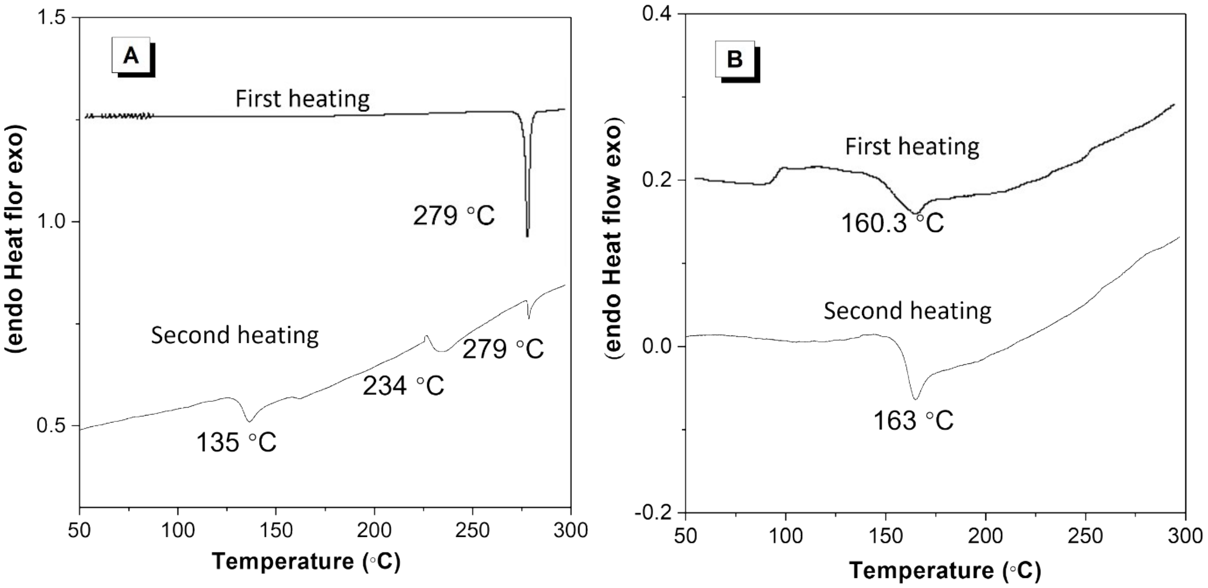

Figure S28. DSC thermograms of (A) BT-2ATPE and (B) BT-2NATPE recorded during the first and second heating cycles under nitrogen at a heating rate of 10 °C/min.

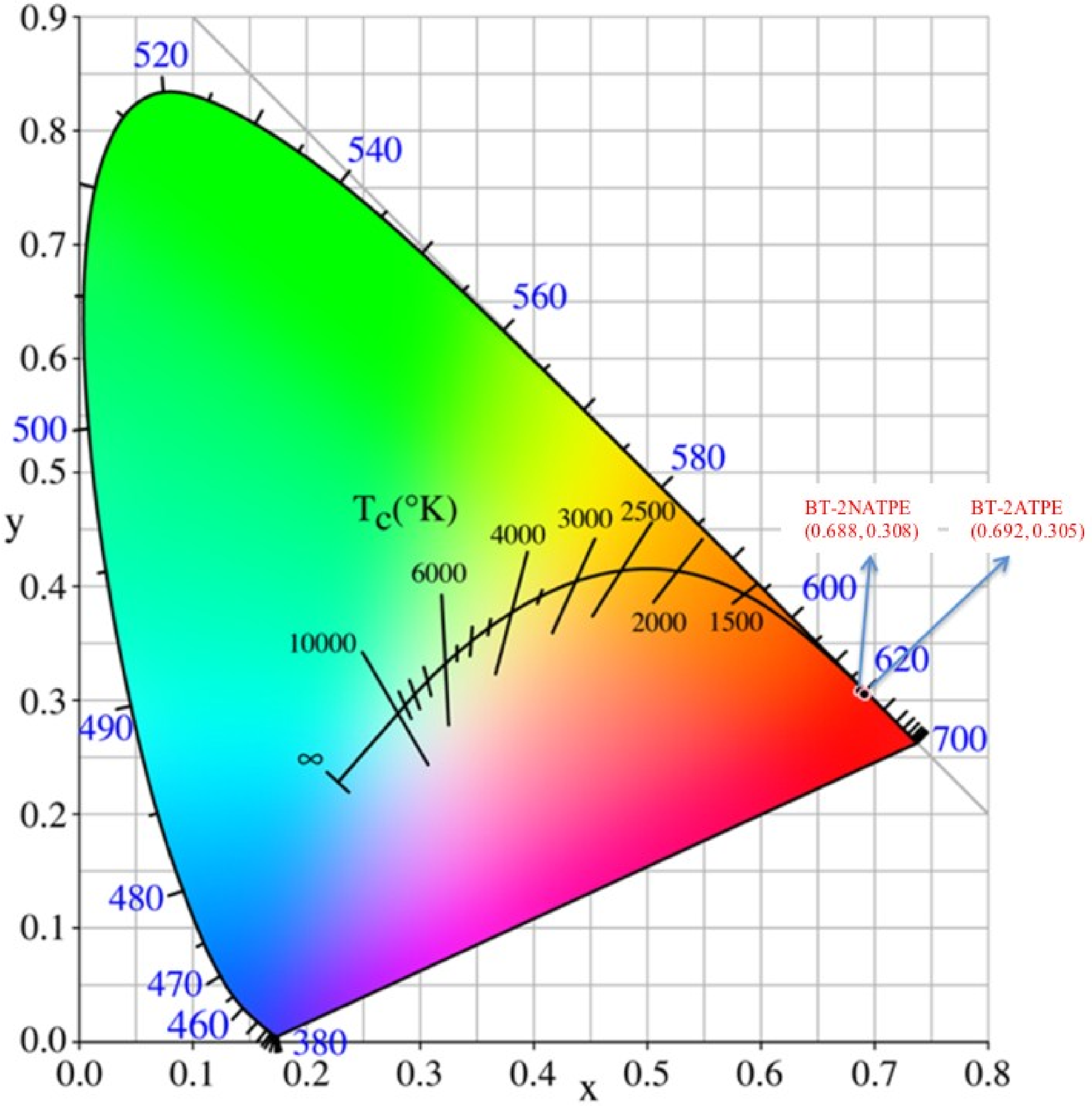

Figure S29. CIE coordinates of BT-2ATPE and BT-2NATPE

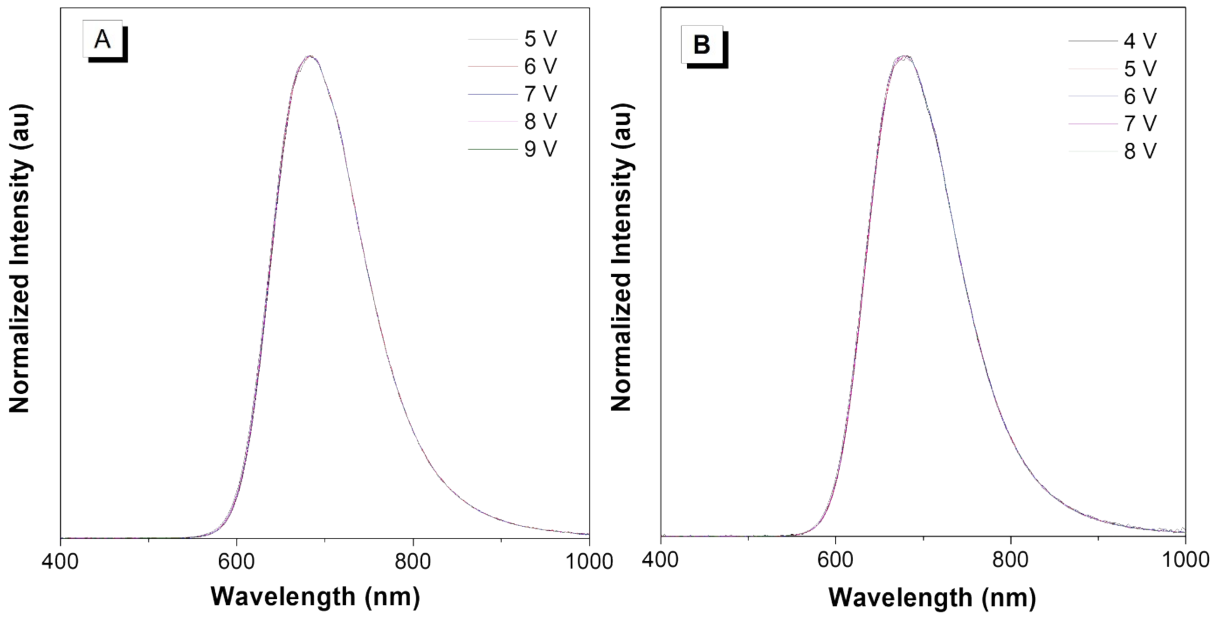

Figure S30. EL spectra of (A) BT-2ATPE and (B) BT-2NATPE under different voltages.

Table S1. Optical properties of BT-ATPE-Ph, BT-ATPE-Py and BT-NATPE-BA.

	λ_{abs} (nm)	λ_{em} (nm)	
		Soln	Film
BT-ATPE-Ph	350, 480	620	635
BT-ATPE-Py	350, 490	615	660
BA-NATPE-BA	345, 485	625	650

Table S2. EL performance of BT-2ATPE and BT-2NATPE.[a)]

	V_{on} (V)	L_{max} (cd/m^2)	λ_{em} (nm)	R_{max} (mW•Sr^{-1}•m^{-2})	CIE (5 V)	EQE (%) at J (mA•cm^{-2})		
						Max	1	10
BT-2ATPE	4.2	251	684	5772	(0.692, 0.305)	1.73	1.72	1.44
BT-2NATPE	4.2	259	682	4692	(0.688, 0.308)	1.43	1.43	1.19
